# Supplementary material for: Functional maturation of enteric neurons derived from human induced pluripotent stem cells
Source: Stem Cell Reports. 2026 Jun 4;21(7):102942. doi: 10.1016/j.stemcr.2026.102942 (PMC13385724; doi:10.1016/j.stemcr.2026.102942)
Supplement: Document S1. Figures S1–S6 [file mmc1.pdf]

Stem Cell Reports, Volume *21*

Supplemental Information

Functional maturation of enteric neurons derived from human induced pluripotent stem cells

Eve S. Rowland, Maciej Daniszewski, Caio Seguin, Maria A. Di Biase, Gunes S. Yildiz, Atefeh Namipashaki, Alice Pébay, Faranak Fattahi, Lincon A. Stamp, and Marlene M. Hao

SUPPLEMENTARY INFORMATION

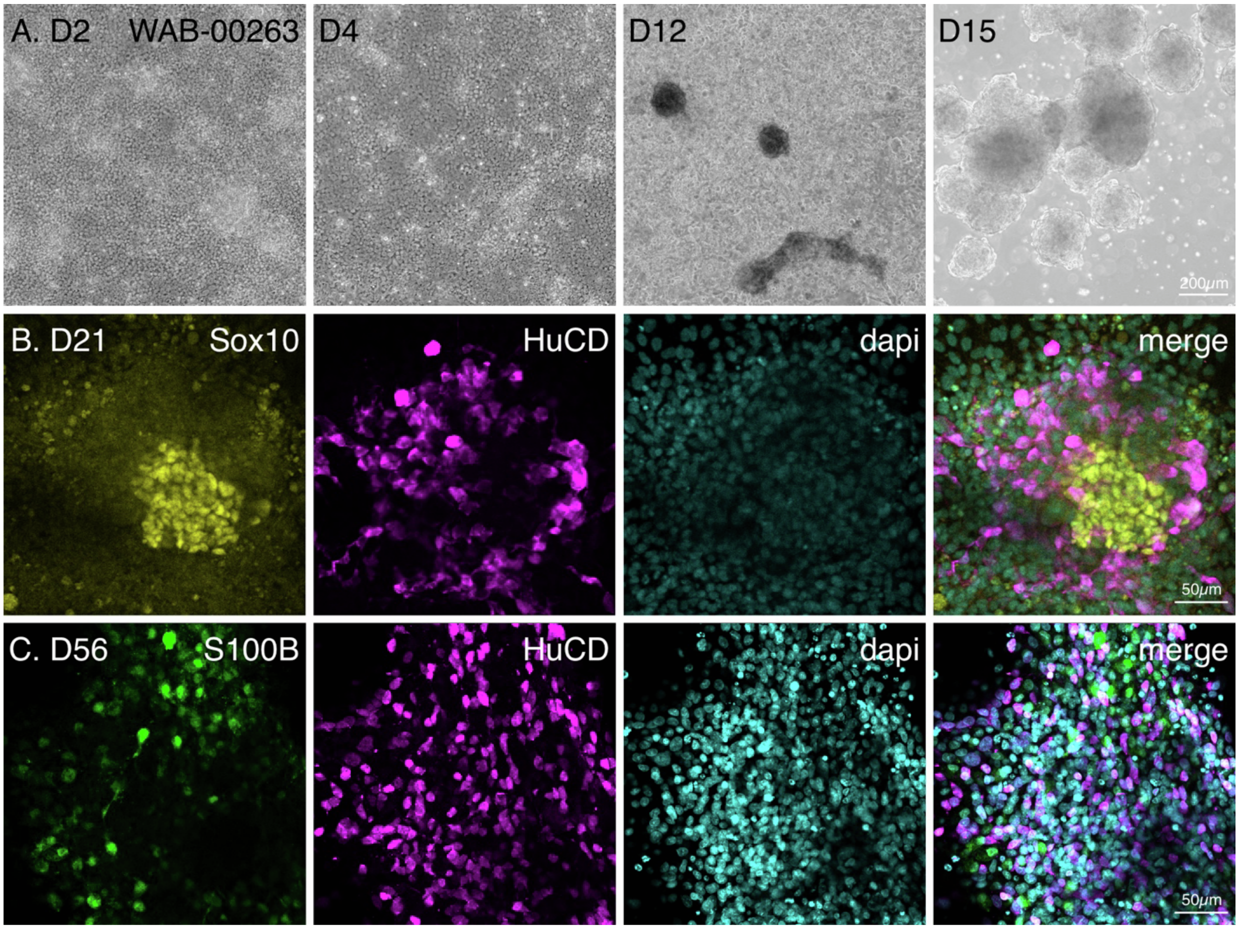

Figure S1: Representative images of differentiation of iPSC-derived ENS for WAB-00263. ***A:*** Representative brightfield images iPSCs at different days of differentiation. ***B:*** iPSCs at D21 of differentiation with immunohistochemistry performed against SOX10 and HuCD. ***C:*** D56 cells with immunohistochemistry against S100B and HuCD. Scale bar = 50 μm.

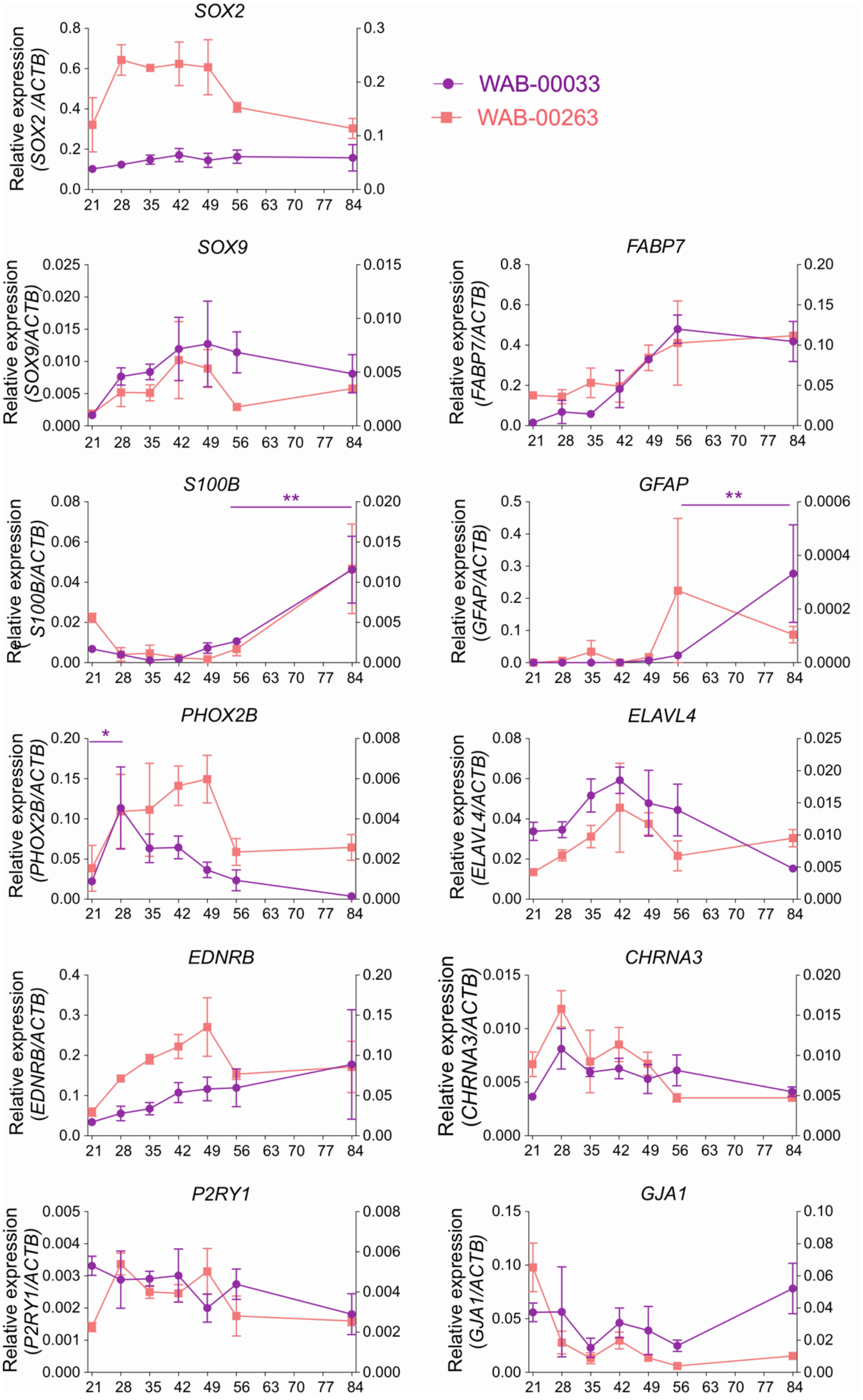
WAB-00033
WAB-00263
SOX2
Relative expression
(SOX2 /ACTB)
SOX9
Relative expression
(SOX9/ACTB)
FABP7
Relative expression
(FABP7/ACTB)
S100B
Relative expression
(S100B/ACTB)
**
GFAP
Relative expression
(GFAP/ACTB)
**
PHOX2B
Relative expression
(PHOX2B/ACTB)
*
ELAVL4
Relative expression
(ELAVL4/ACTB)
EDNRB
Relative expression
(EDNRB/ACTB)
CHRNA3
Relative expression
(CHRNA3/ACTB)
P2RY1
Relative expression
(P2RY1/ACTB)
GJA1
Relative expression
(GJA1/ACTB)
21 28 35 42 49 56 63 70 77 84

Figure S2: Quantitative analysis of expression levels of genes typically expressed during ENS development. Including key neural crest genes (*SOX2*, *SOX9*, *EDNRB*, *PHOX2B*); markers of glial differentiation (*FABP7*, *S100B*, *GFAP*), enteric neurons (*ELAV4*), and genes important in ENS communication (*CHRNA3*, *P2RY1*, *GJA1*), in both WAB-00033 *(purple)* and WAB-00263 *(orange)* cell lines over the time-course of differentiation. Statistically significant changes were observed for glial markers *S100B* and *GFAP* between D56 and D84, and also *PHOX2B* between D21 and D28. Two-way ANOVA followed by Tukey's post hoc test, $^{**}p < 0.01$, $N = 3$ for each cell line.

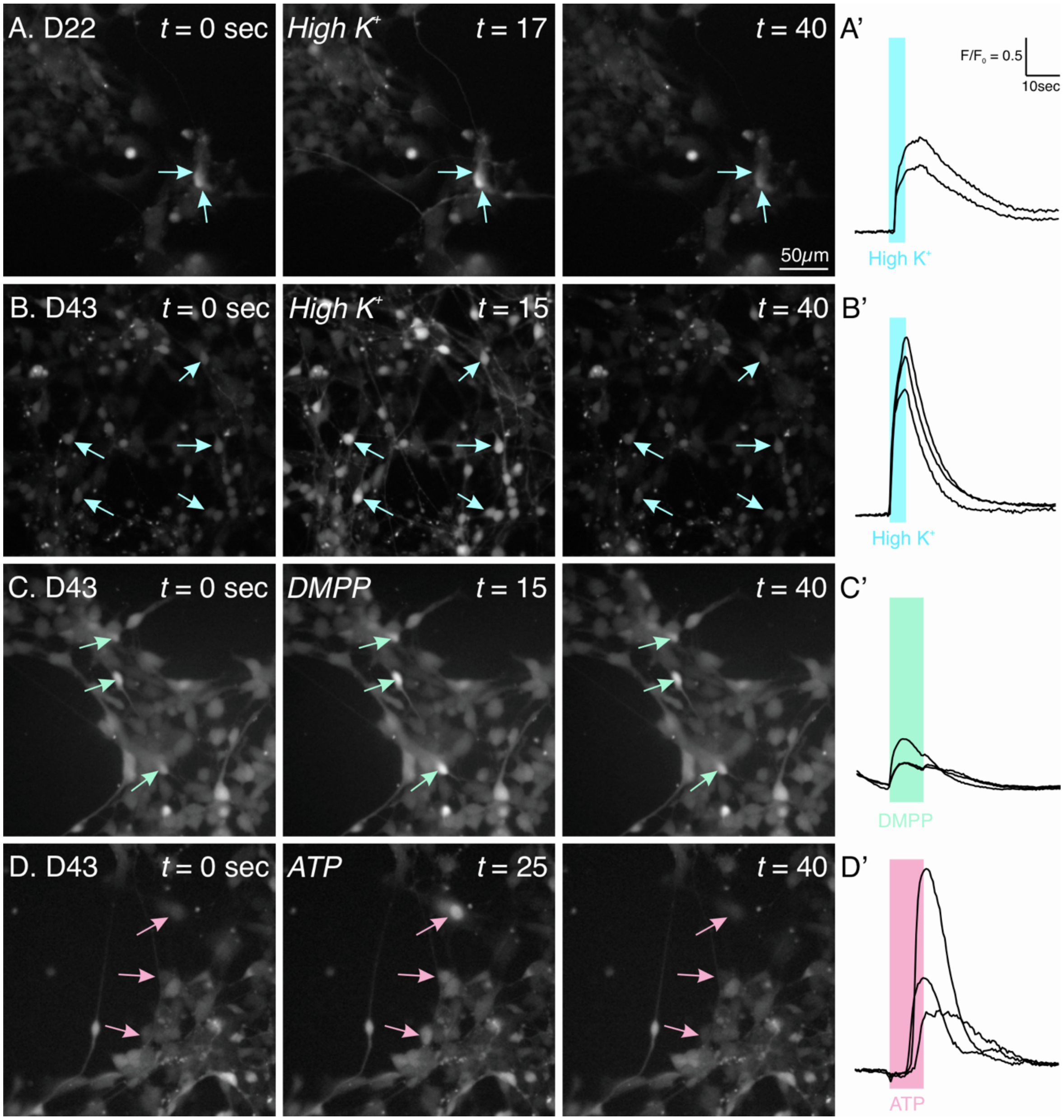

Figure S3: Live Ca^{2+} imaging of iPSC-derived ENS from WAB-00263 in response to depolarisation and neurotransmitter agonists. ***A-B:*** Fluorescence images of Fluo-4 AM loaded cells at D22 ***(A)*** and D43 ***(B)*** at baseline (T = 0 sec), then following High K^+ stimulation (T = 15-17) and (T = 40), with active cells highlighted by arrows. Scale bar: 50 µm. Representative traces of Ca^{2+} response to High K^+ stimulus shown in *A'* and *B'*. Changes in $[Ca^{2+}]_i$ are shown by fluorescence intensity normalised to the baseline fluorescence (F/F_0). Coloured boxes show the duration of High K^+ stimulation from T = 10 – 15 seconds. Vertical scale bar: F/F_0 = 0.5; horizontal scale bar: T = 10 seconds. ***C-D:*** Fluorescence images of Fluo-4 AM loaded cells at D43 at baseline (T = 0 sec), following neurotransmitter agonist stimulation (T = 20) and (T = 40). Scale bar: 50 µm. Representative traces of Ca^{2+} response to DMPP stimulus ***(C,C')*** and ATP stimulus ***(D,D')***.

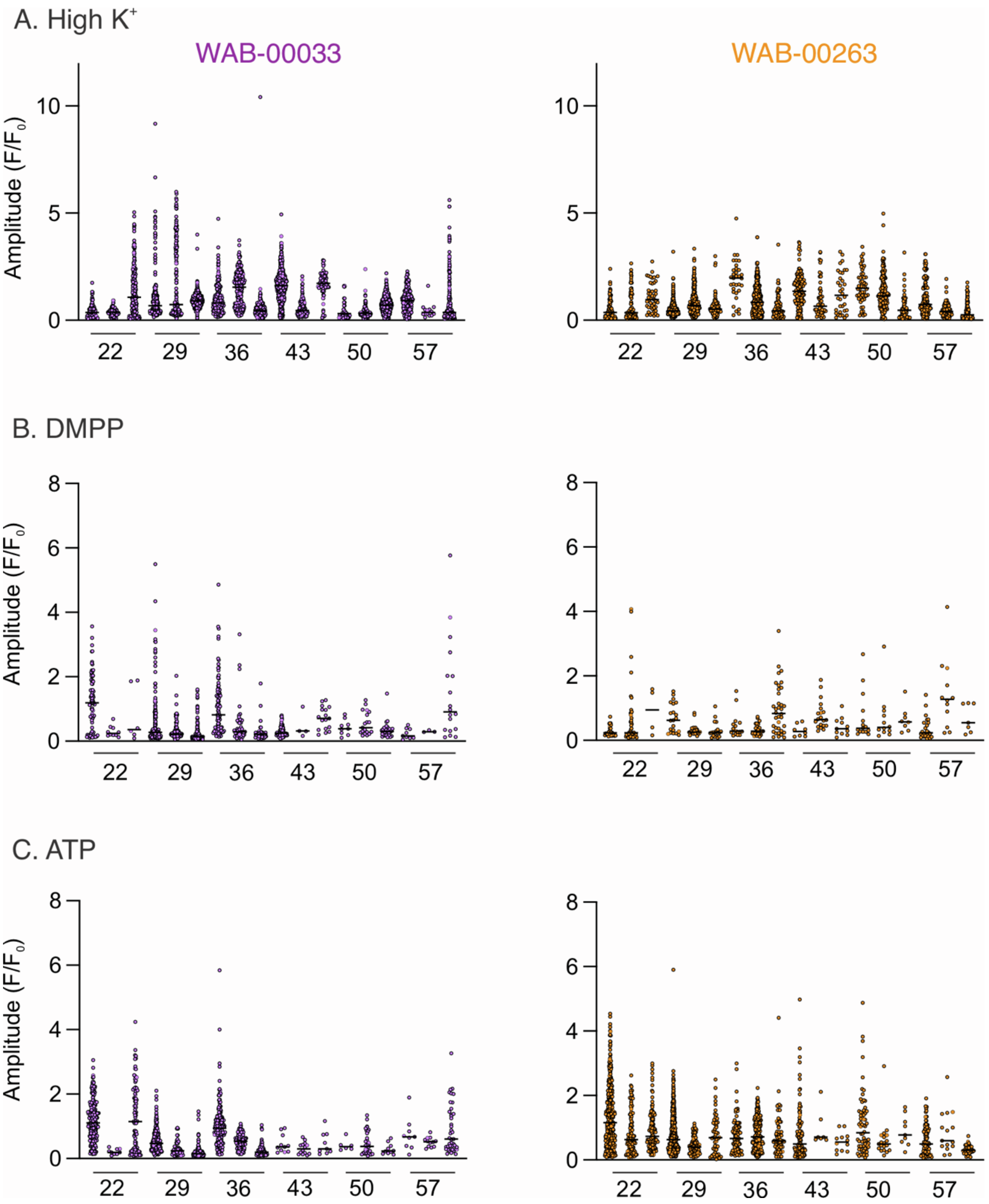

Figure S4: Quantification of evoked $[Ca^{2+}]_i$ transients (F/F_0) in response to High K^+ ***(A)***, DMPP ***(B)*** and ATP ***(C)***, showing individual biological replicates for each cell line: WAB-00033 and WAB-00263. Each data point represents an individual cell, with population means also indicated.

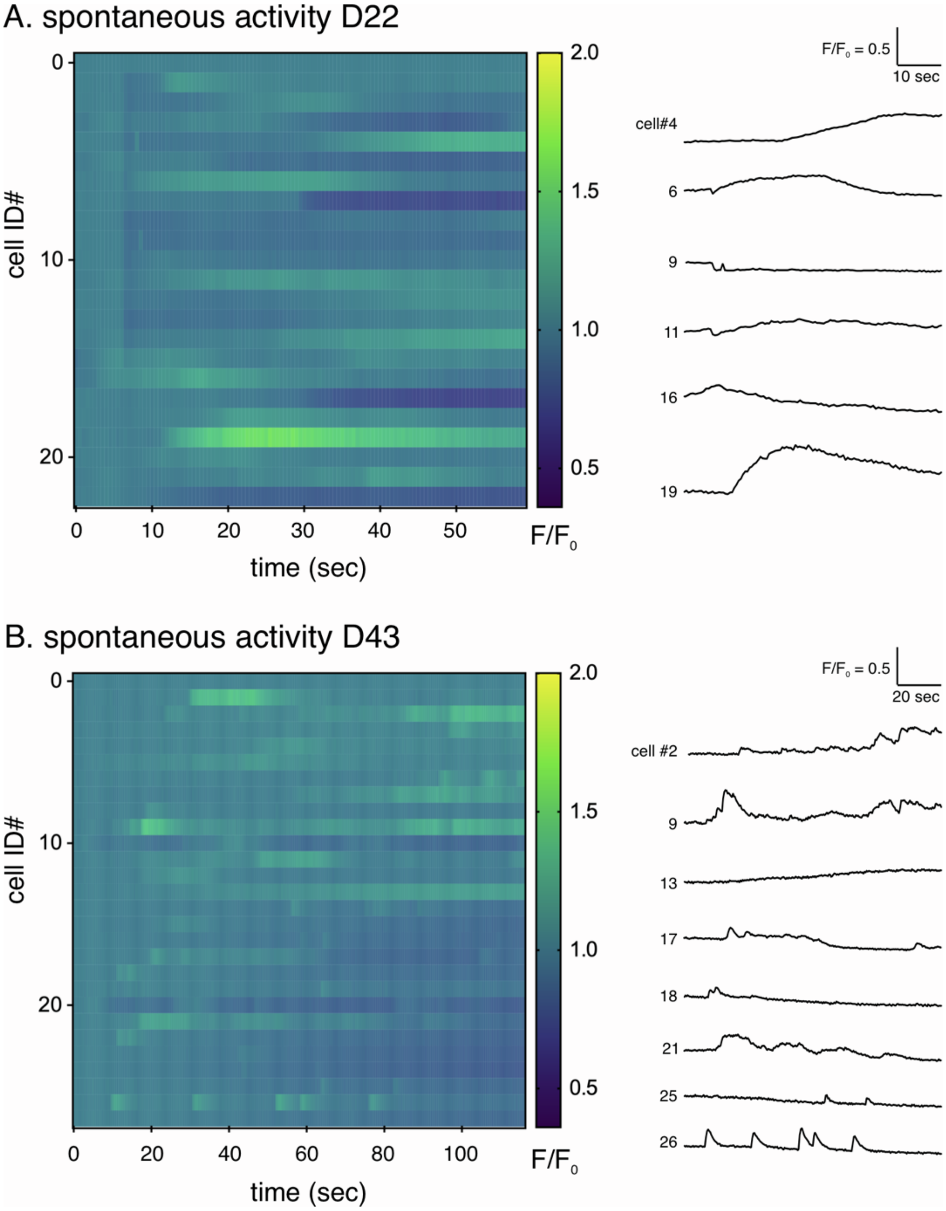

Figure S5: Spontaneous $[Ca^{2+}]_{i,}$ analysis at D22 ***(A)*** and D43 ***(B)***. Heatmap of changes in $[Ca^{2+}]_{i,}$ with fluorescence intensity normalised to the baseline fluorescence F/F_0. Representative traces of spontaneous Ca^{2+} transients from individual cells shown on right. Some spontaneous neuronal $[Ca^{2+}]_{i,}$ transients were present at both D22 and D43, however, activity in individual cells was generally infrequent.

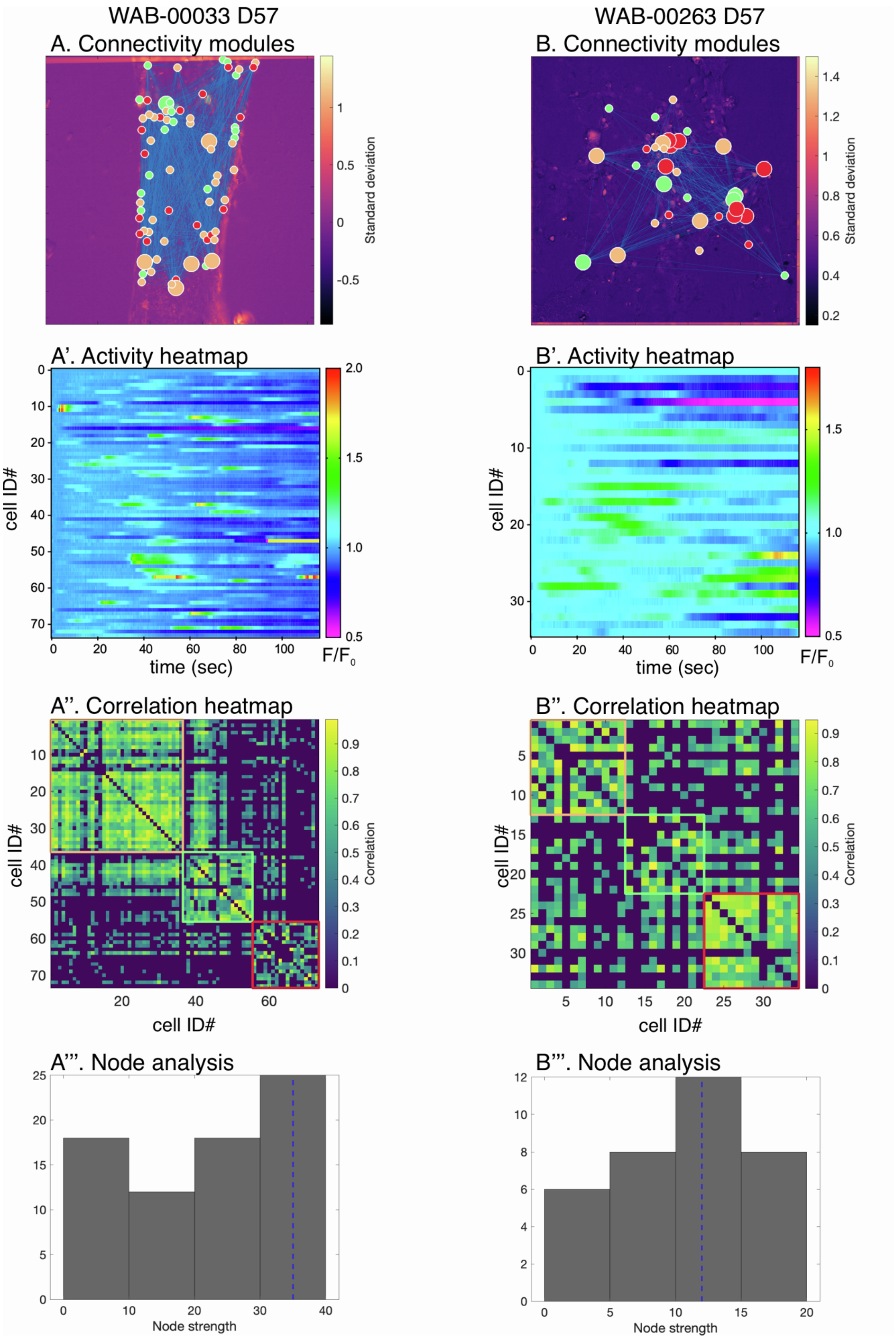

WAB-00033 D57
A. Connectivity modules
Standard deviation
A'. Activity heatmap
cell ID#
time (sec)
F/F_0
A''. Correlation heatmap
cell ID#
Correlation
A'''. Node analysis
Node strength
WAB-00263 D57
B. Connectivity modules
Standard deviation
B'. Activity heatmap
cell ID#
time (sec)
F/F_0
B''. Correlation heatmap
cell ID#
Correlation
B'''. Node analysis
Node strength

Figure S6: Graph theory analysis of $[Ca^{2+}]_i$ network activity at D57 in two separate recordings, one from WAB-00033 (*left, A-A'''*) and one from WAB-00263 (*right, B-B'''*). ***A,B:*** Connectivity modules for each recording were calculated, reconstructed neural network based on 'graph theory' with blue dots representing nodes and blue lines representing edges superimposed on a heatmap of the calcium signals (standard deviation across the time series). Nodes are colour-coded according to network modules. ***A',B':*** Heatmap of $[Ca^{2+}]_i$, activity in individual cells, showing fluorescence intensity after normalisation to baseline fluorescence (F/F_0). ***A'',B'':*** Correlation matrix representation of the neural network, where both the x and y axes correspond to the nodes (cell ID#s), and each element represents the strength of connectivity (correlation value) between pairs of nodes. ***A''',B''':*** Histogram of the degree of each node. The blue dashed line denotes the threshold applied to define 'hub nodes' with high connectivity depicted in *(A,B)*.
