# Supplementary material for: Functional maturation of enteric neurons derived from human induced pluripotent stem cells
Source: Stem Cell Reports. 2026 Jun 4;21(7):102942. doi: 10.1016/j.stemcr.2026.102942 (PMC13385724; doi:10.1016/j.stemcr.2026.102942)
Supplement: Document S2. Article plus supplemental information [file mmc7.pdf]

STEM CELL REPORTS
Article
OPEN ACCESS

Functional maturation of enteric neurons derived from human induced pluripotent stem cells

Eve S. Rowland,[1] Maciej Daniszewski,[1] Caio Seguin,[4] Maria A. Di Biase,[1,4,5] Gunes S. Yildiz,[1] Atefeh Namipashaki,[1] Alice Pébay,[1,2] Faranak Fattahi,[3] Lincon A. Stamp,[1,*] and Marlene M. Hao[1,6,*]
[1]Department of Anatomy and Physiology, the University of Melbourne, Melbourne, VIC, Australia
[2]Department of Surgery, Royal Melbourne Hospital, The University of Melbourne, Melbourne, VIC, Australia
[3]Department of Cellular and Molecular Pharmacology, University of California, San Francisco, San Francisco, CA, USA
[4]Department of Psychiatry, the University of Melbourne, Melbourne, VIC, Australia
[5]Department of Psychiatry, Brigham and Women's Hospital, Harvard Medical School, Boston, MA, USA
[6]Lead contact
*Correspondence: lstamp@unimelb.edu.au (L.A.S.), hao.m@unimelb.edu.au (M.M.H.)
https://doi.org/10.1016/j.stemcr.2026.102942

SUMMARY

The enteric nervous system (ENS) plays a crucial role in regulating gastrointestinal function, including motility, secretion, and absorption. Enteric neurons and glia derived from human induced pluripotent stem cells (iPSCs) have vast applications in disease modeling, drug screening, and cell replacement therapy, yet our understanding of their functional maturation remains limited. Using a defined protocol for the generation of iPSC-derived enteric neurons, we examined neuronal differentiation with immunohistochemistry, gene expression analysis, and cellular-resolution calcium (Ca^{2+}) imaging from day (D)21 to D57 of differentiation. By D21, cells expressing pan-neuronal markers were present, and at D22 the cultures were responsive to K^+-induced depolarization as well as neurotransmitter receptor agonists, with maturation of responses to high K^+ depolarization during the differentiation period. In addition, the development of spontaneous neuronal network activity was observed at 2 months post-differentiation. Together these findings provide a timeline of the functional maturation of iPSC-derived ENS cells.

INTRODUCTION

The enteric nervous system (ENS) is the most complex division of the peripheral nervous system, encompassing all neurons and glia intrinsic to the gut wall (Furness, 2012). Enteric neurons and glia form a dedicated neurocircuitry responsible for controlling intestinal motility, mucosal blood flow, nutrient absorption and epithelial secretion, and engage in bidirectional communication with the brain via the gut brain axis. During development, the ENS is derived from the neural crest (Le Douarin and Teillet, 1973; Yntema and Hammond, 1954) and begins to show signatures of neural activity as early as the mid-embryonic stage in mice and humans (Hao et al., 2011; McCann et al., 2019). Studies in mice have shown that neural activity in developing enteric neurons changes from the fetal period into adulthood, with shifts in response patterns, signal strength, and duration (Foong et al., 2012; Hao et al., 2011; 2012). This establishment and maturation of neural activity throughout the embryonic and early postnatal periods is crucial to the development of the functional networks essential for gut physiology (Hao et al., 2016).

Disruptions to ENS development can lead to enteric neuropathies including Hirschsprung disease, which results from altered migration, differentiation, or survival of enteric neural crest cells, leading to aganglionosis and impaired motility in affected bowel segments (Heuckeroth, 2018; McKeown et al., 2013; Montalva et al., 2023). While life-saving, the current gold standard surgical treatment for Hirschsprung disease still leaves over 50% of patients with life-long complications, including incontinence, chronic constipation, and enterocolitis (inflammation of the bowel) (Montalva et al., 2023). Advances in diagnosis and surgical techniques have improved patient outcomes, however there is currently growing interest in the use of stem cell-based therapies for restoring ENS function and improving long-term outcomes for patients (Burns et al., 2016).

With the advent of pluripotent stem cell (PSC) technology, much research has focused on generating enteric neural cells from human PSCs for cell transplantation. Early studies centered on generating neural crest cells, particularly vagal neural crest cells, the precursor cells from which the majority of enteric neurons and glia differentiate (Fattahi et al., 2016; Hackland et al., 2017; Lee et al., 2007; 2010; Lee et al., 2007; 2010). More recently, protocols have been developed to specifically generate enteric neurons from PSCs, utilizing signaling pathways that mimic the developmental cues of the neural crest lineage (Barber et al., 2019; Fan et al., 2023; Frith et al., 2020; Majd et al., 2025). These protocols produce cultures rich in various subtypes of enteric neurons, with populations of enteric glia reported to emerge with prolonged differentiation. Studies using PSC-derived ENS cells have highlighted their capacity to engraft into the murine gut, suggesting potential in the treatment of enteric neuropathies (Fan et al., 2023; Fattahi et al., 2016; Majd et al., 2025). Despite their

Published by Elsevier Inc. on behalf of International Society for Stem Cell Research.
This is an open access article under the CC BY license (http://creativecommons.org/licenses/by/4.0/).

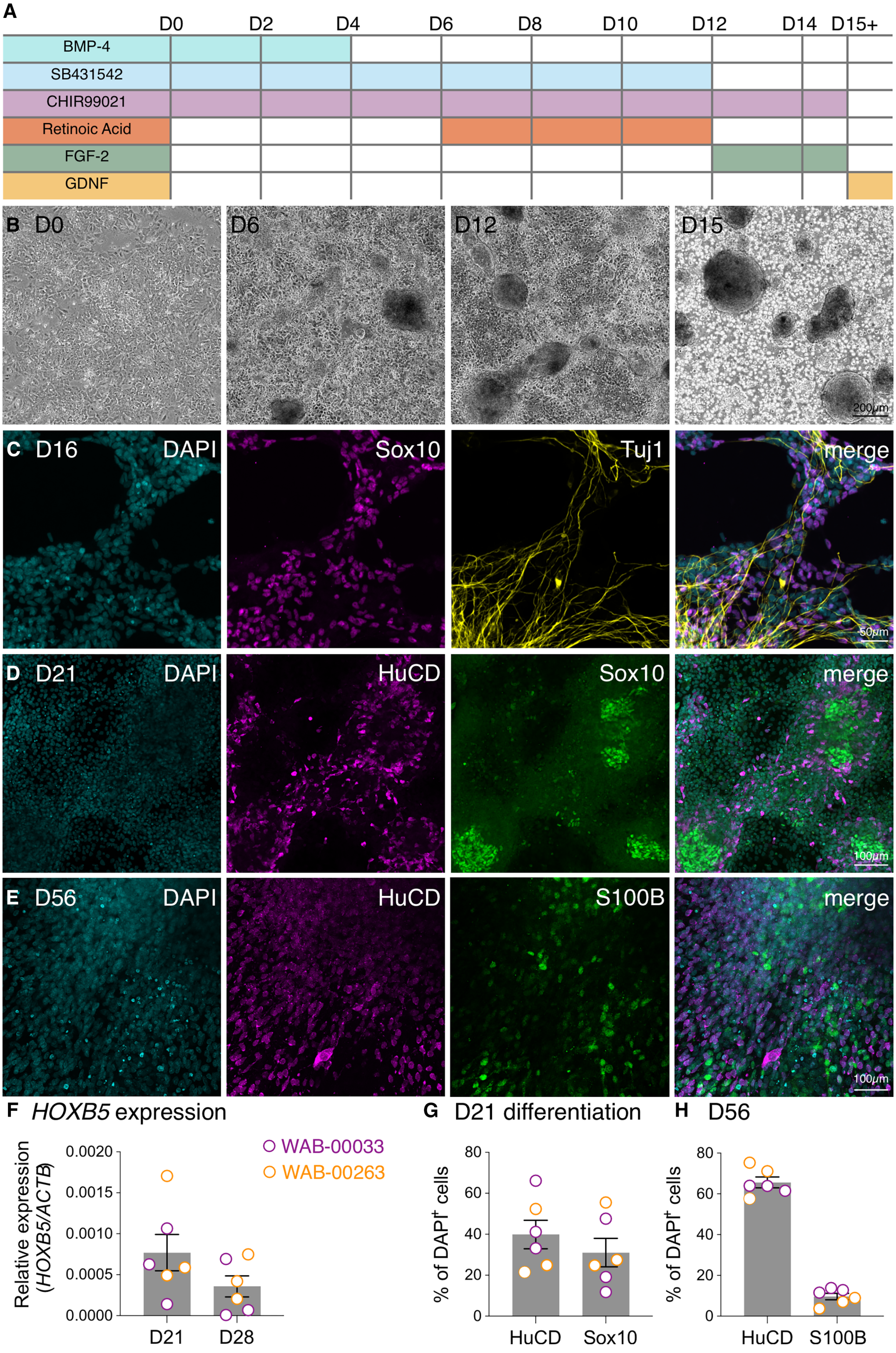

(legend on next page)

potential therapeutic application, the functional characteristics of PSC-derived enteric neurons remain largely unexplored. Understanding how PSC-derived enteric neurons differentiate and acquire function is crucial, particularly in determining how these cells form neural networks and whether they accurately recapitulate *in vivo* physiology.

In this study, we examined changes in the Ca^{2+} dynamics of human induced PSC (iPSC)-derived enteric neurons during *in vitro* maturation at the single-cell level. Live cell calcium imaging has emerged as an important tool for investigating the development and function of the enteric nervous system and is often used as a proxy for neuronal activity (Boesmans et al., 2013). In addition, we investigated the development of neuronal network connections through network construction and analysis by graph theory (Bullmore and Sporns, 2009). The use of iPSC-derived enteric neurons has vast applications in disease modeling, drug screening, and cell replacement therapies, but many important questions about cellular identity, maturation, and function remain unanswered. Improved knowledge of the developmental and functional properties of enteric neurons derived from iPSCs is a critical next step in validating their relevance and utility as biological models.

RESULTS

Differentiation of iPSCs into ENS lineages

In this study, we used a previously established protocol for the derivation of enteric neuron lineages from iPSCs (Barber et al., 2019) (Figure 1A). Two iPSC lines (WAB-00033 and WAB-00263) were previously generated as control lines and have been characterized for pluripotency (Daniszewski et al., 2022). iPSCs were differentiated to a cranial neural crest cell fate by the timed delivery of bone morphogenic protein 4 (BMP-4) and small molecule inhibitors of the Activin/Nodal (SB431542) and GSK3β (CHIR99021) signaling pathways. Subsequently, cells were transitioned to a vagal/enteric neural crest cell identity by addition of the caudalizing factor retinoic acid to the culture medium. Cultures were maintained in the presence of fibroblast growth factor 2 (FGF-2) to enrich for vagal neural crest cells, followed by glial-derived neurotrophic factor (GDNF) to differentiate toward an enteric neuron lineage (Figure 1B). At day (D)16 of differentiation, immunohistochemistry was performed and neural crest progenitor identity confirmed by presence of SOX10 (Figure 1C). Neurite projections could also be observed extending from the cell soma from as early as D16 (Figure 1C). By D21, cells appeared to self-aggregated into lattice-like structures, which were interconnected by neurite bundles (Figures 1D and S1B). Neuronal differentiation was examined using immunohistochemistry against the pan-neuronal marker, HuCD. At D21, 39.90 ± 6.96% of DAPI+ cells were HuCD-immunoreactive, with a further 31.07 ± 6.92% expressing Sox10. By D56, the percentage of DAPI+ cells expressing HuCD increased to 65.62 ± 2.64. Glial differentiation appeared at later stages of differentiation, with 9.67 ± 1.56% of DAPI+ expressed the mature enteric glia marker S100B at D56 (Figures 1E–1H; Figure S1C). Overall, there were marked similarities between the differentiation trajectories of the two iPSC lines, however, each cell line was analyzed separately as data were collected sequentially through each differentiation replicate.

Gene expression signatures mirror enteric nervous system development

To confirm the differentiation of enteric nervous system lineages, we performed temporal gene expression analysis for a panel of genes expressed during ENS development using digital PCR (dPCR) from D21 to D84 days of differentiation. Gene expression levels were normalized to the house keeping gene β-actin (*ACTB*). To confirm vagal neural crest identity, *HOXB5* expression was investigated at early stages of differentiation and found to be present at both D21 and D28 (Figure 1F). Enteric neural crest cell identity was characterized by expression of a variety of markers, including paired-like homeobox 2B (*PHOX2B*), the endothelin receptor B (*EDNRB*), and members of the Sox family of transcription factors, including *SOX2* and *SOX9*. These genes are also expressed in the derivatives of enteric neural crest

Figure 1. Differentiation of iPSC-derived ENS

(A) Schematic diagram of growth factor and small molecule-mediated ENS differentiation protocol for enteric nervous system lineages.

(B) Representative brightfield images of ENS differentiation from D0 to D16 of differentiation from WAB-00033. Scale bars, 200 μm.

(C) iPSC differentiation at D16 with immunohistochemical staining of the enteric neural crest marker SOX10, and neuronal class III b-tubulin (Tuj1). Scale bars, 50 μm.

(D) iPSC differentiation at D21, showing immunohistochemical staining for SOX10 and the pan-neuronal marker, HuCD. Scale bars, 100 μm.

(E) iPSC differentiation at D56, many HuCD neurons are present and a small population of glial cells (S100B+). Scale bars, 100 μm.

(F) Gene expression analysis of the vagal neural crest marker *HOXB5* at D21 and D28.

(G) Quantification of HuCD-immunoreactive and Sox10-immunoreactive cells out of the total number of DAPI+ cells at D21 of differentiation.

(H) Quantification of HuCD-immunoreactive and S100B-immunoreactive cells out of the total number of DAPI+ cells at D56.

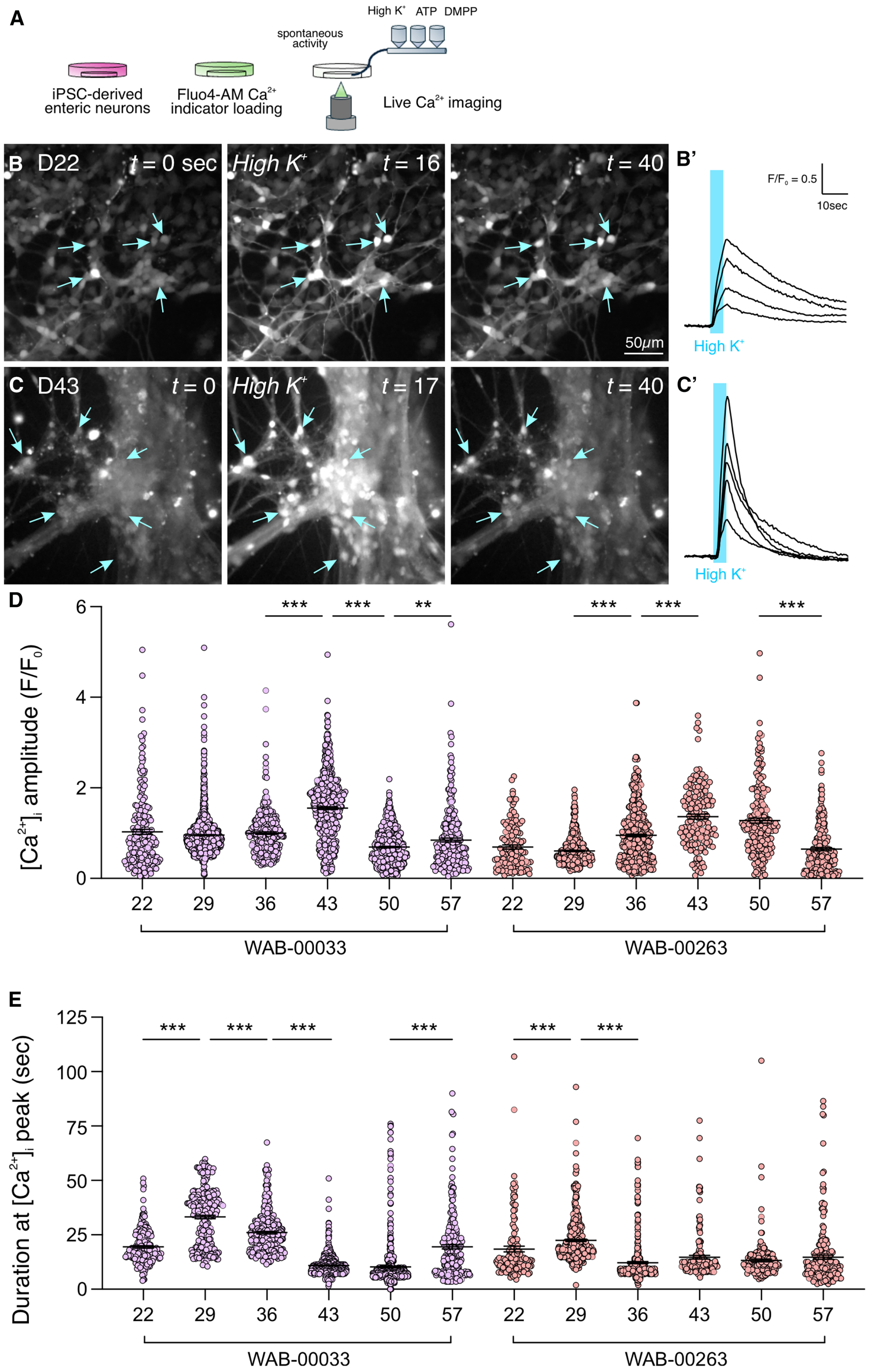

(legend on next page)

cell precursors, with subsets of enteric neurons expressing *PHOX2B* and *EDNRB*. Expression of *PHOX2B*, *EDNRB*, *SOX2*, and *SOX9* was detected in differentiated cultures from D21, indicative of the generation of enteric neural crest cell lineages in both cell lines (Figure S2). We also examined expression of the pan-neuronal marker HuD which is encoded by the *ELAVL4* gene. *ELAVL4* expression was detected at all stages of differentiation, confirming neuronal lineage commitment (Figure S2).

As populations of enteric glia have previously been shown to emerge in enteric cultures after prolonged differentiation, we examined the temporal expression of canonical enteric glial cell marker genes, including *FABP7* (which encodes the glial precursor marker, B-FABP), *S100B* (encoding the calcium binding protein S100β), and *GFAP* (glial fibrillary acidic protein). The maturity of enteric glia can be distinguished by expression of these genes, with immature committed enteric glial progenitors expressing *FABP7* but not *S100B* and *GFAP* and mature enteric glia downregulating *FABP7* expression and upregulating *S100B* and *GFAP* expression (Young et al., 2003). Expression of *FABP7* and *S100B* was detected at all developmental time points, indicating that a population of committed enteric glial progenitors with varying degrees of maturity are present at early stages of differentiation. *S100B* and *GFAP* expression generally remained low until D56, suggestive of increasing maturation of glial progenitors with prolonged culture (Figure S2).

We also examined the expression of genes involved in enteric neuron and glia communication in our iPSC model of the ENS. Acetylcholine is one of the major excitatory neurotransmitters of the ENS, and many subtypes of enteric neurons communicate through cholinergic nicotinic receptors. We therefore investigated expression of *CHRNA3*, which encodes the nicotinic receptor subunit α3, one of the main subunits expressed in the mature mammalian ENS (Foong et al., 2015). We also investigated the expression of *P2RY1* and *GJA1* which encode the P2Y1 receptor and gap junction alpha 1 (GJA1, also known as connexin-43) proteins, respectively. Purinergic P2Y1 receptors are expressed by both enteric neurons and enteric glial cells. P2Y1 receptors on enteric glia are important for neuron-to-glia communication (Hendler et al., 2024). GJA1 is an important gap junction protein, which is expressed in the developing ENS and by enteric glia (McClain et al., 2014). Expression of *CHRNA3*, *P2RY1* and *GJA1* were detected at D21, and maintained throughout the differentiation period, suggesting that components of the machinery necessary for intercellular communication between enteric neurons and glia were present (Figure S2).

Functional development of iPSC-derived enteric neurons

Given gene expression and immunohistochemical data indicated robust generation of enteric neurons, we sought to define the functional maturation of the cell population over the course of the first 2 months of differentiation. Live cell Ca^{2+} imaging was performed every 7 days from D22 of differentiation and changes in intracellular calcium concentration $[Ca^{2+}]_i$ examined in response to depolarization using high K^+ (75 mM) and neurotransmitter receptor agonists (Figure 2A). Enteric neurons were readily identifiable in culture based on their distinctive cell morphology, with cultures consisting of clusters of spherical cell bodies from which neurite projections extended.

To examine the electrical properties of neuronal cultures, we stimulated differentiated cells with high K^+. Elevated extracellular levels of K^+ depolarize the neuronal membrane and induce robust Ca^{2+} transients in cultures of primary enteric neurons (Hao et al., 2011). For each $[Ca^{2+}]_i$ response, the amplitude ($\Delta F/F_0$) and duration (at 50% amplitude) were measured. Individual responding cells were examined at all developmental time-points. The temporal profile of both $[Ca^{2+}]_i$ amplitude and duration was similar for both cell lines in response to high K^+ stimulation, although some differences were observed (Figures 2B and 2C; Figures S3A and S3B). The amplitude of $[Ca^{2+}]_i$ increased between D22 and D43, with maximum Ca^{2+} responses generated on D43. Beyond D43, there was a decrease in the $[Ca^{2+}]_i$ transients amplitudes at D50 and

Figure 2. Live Ca^{2+} imaging of iPSC-derived ENS

(A) Schematic diagram of live cell Ca^{2+} imaging experimental protocol showing Fluo-4 AM dye loading, experimental conditions for agonist evoked and spontaneous recordings of the iPSC-ENS and data analysis.

(B and C) Fluorescence images of Fluo-4 AM loaded cells from WAB-00033 at D22 (B) and D43 (C) at baseline (T = 0 s), following high K^+ stimulation (T = 16–17). At T = 40, many D22 cells still exhibit elevated fluorescence levels, while most D43 cells have returned to baseline fluorescence. Active cells highlighted by arrows. Scale bars, 50 μm. (B′) and (C′) Representative traces of Ca^{2+} response to high K^+ stimulus. Changes in $[Ca^{2+}]_i$ are shown by fluorescence intensity normalized to the baseline fluorescence (F/F_0). Blue box shows duration of high K^+ stimulus from T = 10–15 s. Vertical scale bars, F/F_0 = 0.5; horizontal scale bars, T = 10 s.

(D) Amplitude of $[Ca^{2+}]_i$ transients ($\Delta F/F_0$) following high K^+ stimulus for the cell lines WAB-00033 (purple) and WAB-00263 (orange).

(E) $[Ca^{2+}]_i$ duration (calculated at 50% of peak amplitude) for the cell lines WAB-00033 (purple) and WAB-00263 (orange) in response to high K^+ stimulation. Each data point represents an individual cell, with population mean ± SEM shown. *N* = 3 differentiation experiments for WAB-00033 and WAB-00263. Two-way ANOVA followed by Tukey's post hoc test. **p* < 0.05, ***p* < 0.01, ****p* < 0.001.

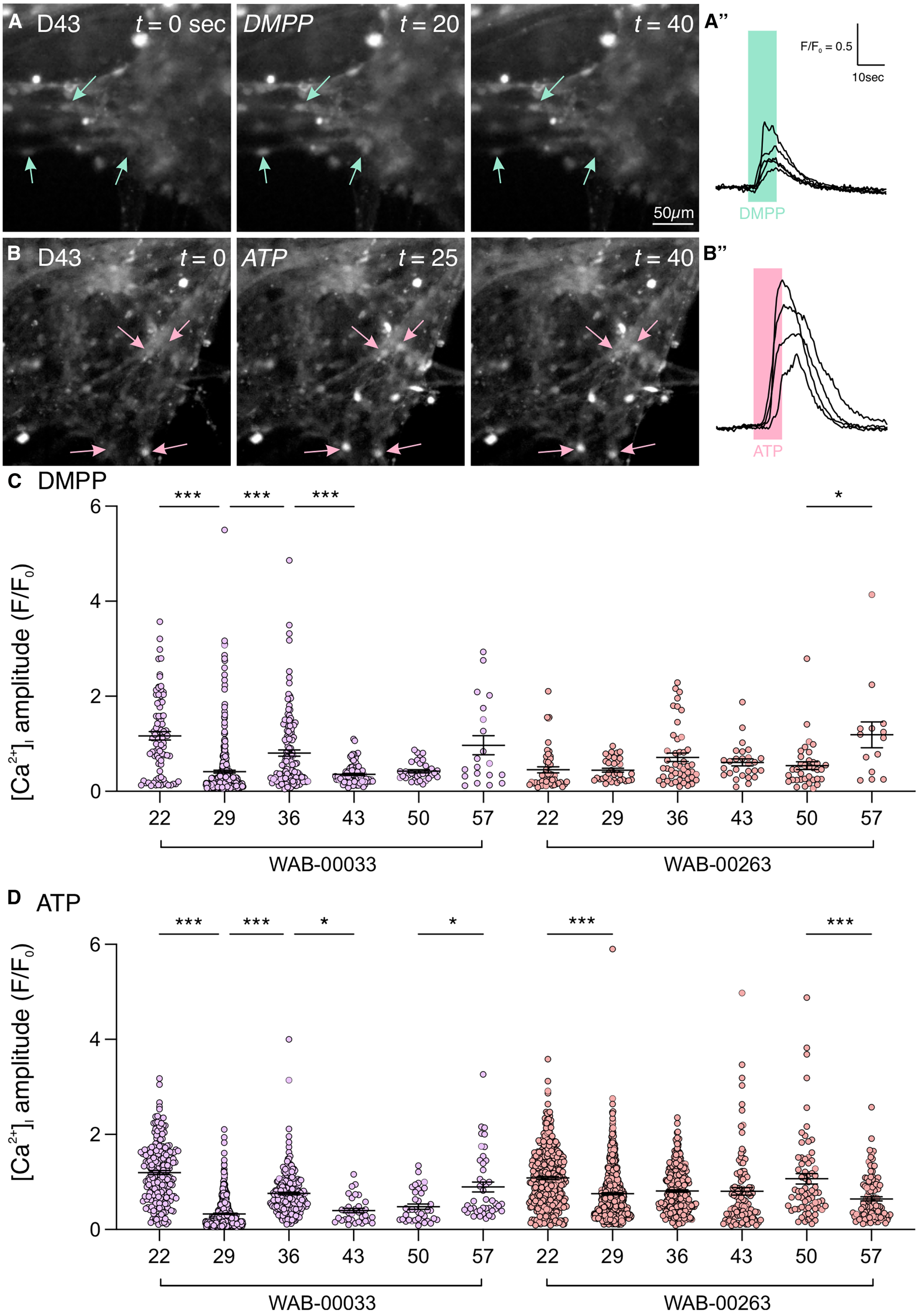

(legend on next page)

D57. While there were some differences between WAB-00033 and WAB-00263, the pattern of increasing and then decreasing $[Ca^{2+}]_i$ transients amplitude through differentiation was apparent for both cell lines. We also observed overlap of data from across individual biological replicates for both cell lines (Figure S4).

$[Ca^{2+}]_{i,}$ transient duration significantly increased between D22 and D29, with maximum duration of Ca^{2+} transients observed on D29 (Figure 2D′). Beyond D29 the duration of the Ca^{2+} transient significantly decreased. Interestingly, in the cell line WAB-00033, the $[Ca^{2+}]_{i,}$ transient duration significantly increased from D50 to D57, which was not observed in WAB-00263. Due to the sheer density of cells in culture, and issues with cell detachment during processing, we were unable to perform post hoc immunohistochemistry to correlate individual cell responses with neuronal and glial markers in this study.

Neurotransmitter agonists induce Ca^{2+} transients

In addition to depolarization-induced Ca^{2+} transients, we assessed neurotransmitter responsiveness to further characterize functional properties of the differentiated neurons (Figures 3A and 3B). The functions of the ENS are regulated by a variety of neurotransmitters. As described above, acetylcholine is the major excitatory neurotransmitter of the ENS, acting via nicotinic receptors on enteric neurons. We examined changes in the amplitude of intracellular $[Ca^{2+}]_i$ in response to the nicotinic acetylcholine receptor (nAChR) agonist, DMPP (10 μM), and found that at all times between D22 and D57 of differentiation, subpopulations of cells responded to DMPP application (Figures 3A and 3C; Figure S3). The amplitude of intracellular Ca^{2+} transients in response to DMPP was similar for both cell lines at each developmental stage, with the exception of D22 where response amplitude was greater in the cell line WAB-00033 compared to WAB-00263 (Figure 3). There was no correlation between the age of cultures and the amplitude of Ca^{2+} transients in response to DMPP stimulus.

We next examined responses to the purinergic receptor agonist adenosine triphosphate (ATP). ATP acts as both an excitatory and inhibitory neurotransmitter in the ENS and facilitates bidirectional communication between enteric neurons and glia (Gomes et al., 2009; Gulbransen and Sharkey, 2009; Gwynne and Bornstein, 2007), and purinergic transmission has been found to be responsible for waves of calcium activity during early ENS development (Hao et al., 2017). In both cell lines, subpopulations of cells were ATP responsive at all stages of differentiation (Figures 3B and 3D; Figure S3). The amplitude of the $[Ca^{2+}]_i$ peak induced by ATP showed some differences at each developmental stage but there was no obvious correlation between the age of differentiated cultures and the amplitude of intracellular Ca^{2+} response following ATP stimulus.

Presence of spontaneous Ca^{2+} oscillations and network activity

The emergence of spontaneous activity is a characteristic feature of nervous system development and spontaneous calcium activity is observed in the developing ENS at the earliest stages of foregut colonization. We performed recordings of spontaneous Ca^{2+} activity in differentiated cultures and found that at all stages of differentiation, cells displaying spontaneous Ca^{2+} oscillations were present, albeit in most cases only a few spontaneously active cells were observed in each recording (Figure S5). At D22, spontaneous Ca^{2+} oscillations were infrequent and of small amplitude and long duration (Figure S5). As differentiation progressed, the frequency and amplitude of spontaneous Ca^{2+} transients appeared to increase, however, very few cells exhibited regular or recurring spontaneous $[Ca^{2+}]_i$ transients at both D22 and D43. At D57, subpopulations of cells with more frequent and repetitive bursts of spontaneous Ca^{2+} activity were observed (Figure 4). These bursts of activity were observed in 50% (3/6) of recordings at D57 of differentiation (2 from WAB-00033 and 1 from WAB-00263), but were not noticeable at earlier ages.

To investigate this further, we constructed a network where nodes represent regions of interest defined by $[Ca^{2+}]_i$ signals, and edges represent the correlation between $[Ca^{2+}]_i$ signals of these regions (Figures 4 and S6). Graph analysis was used to identify modules of synchronous activity across the network (Bullmore and Sporns, 2009; Rubinov and Sporns, 2010; Schröter et al., 2017) (see "STAR Methods"). Modules delineate cell clusters with coherent functional profiles thought to reflect components of specialized information processing (Sporns and Betzel, 2016;

Figure 3. Live Ca^{2+} imaging of iPSC-derived ENS in response to neurotransmitter agonists

(A and B) Fluorescence images of Fluo-4 AM loaded cells from WAB-00033 at D43 at baseline (T = 0 s), following neurotransmitter agonist stimulation (T = 20) and (T = 40). Scale bars, 50 μm. Representative traces of Ca^{2+} response to DMPP stimulus (A′) and ATP stimulus (B′). Changes in $[Ca^{2+}]_i$ are shown by fluorescence intensity normalized to the baseline fluorescence (F/F_0). Colored boxes show the duration of DMPP or ATP stimulation from T = 10–20 s. Vertical scale bars, $F/F_0 = 0.5$; horizontal scale bars, T = 10 s.

(C and D) Amplitude of $[Ca^{2+}]_i$ transients ($\Delta F/F_0$) in response to DMPP stimulus (C) and ATP (D) for the cell lines WAB-00033 (purple) and WAB-00263 (orange). Each data point represents an individual cell, with population mean ± SEM shown. $N = 3$ differentiation experiments for WAB-00033 and WAB-00263. Two-way ANOVA followed by Tukey's post hoc test. $^*p < 0.05$, $^{**}p < 0.01$, $^{***}p < 0.001$.

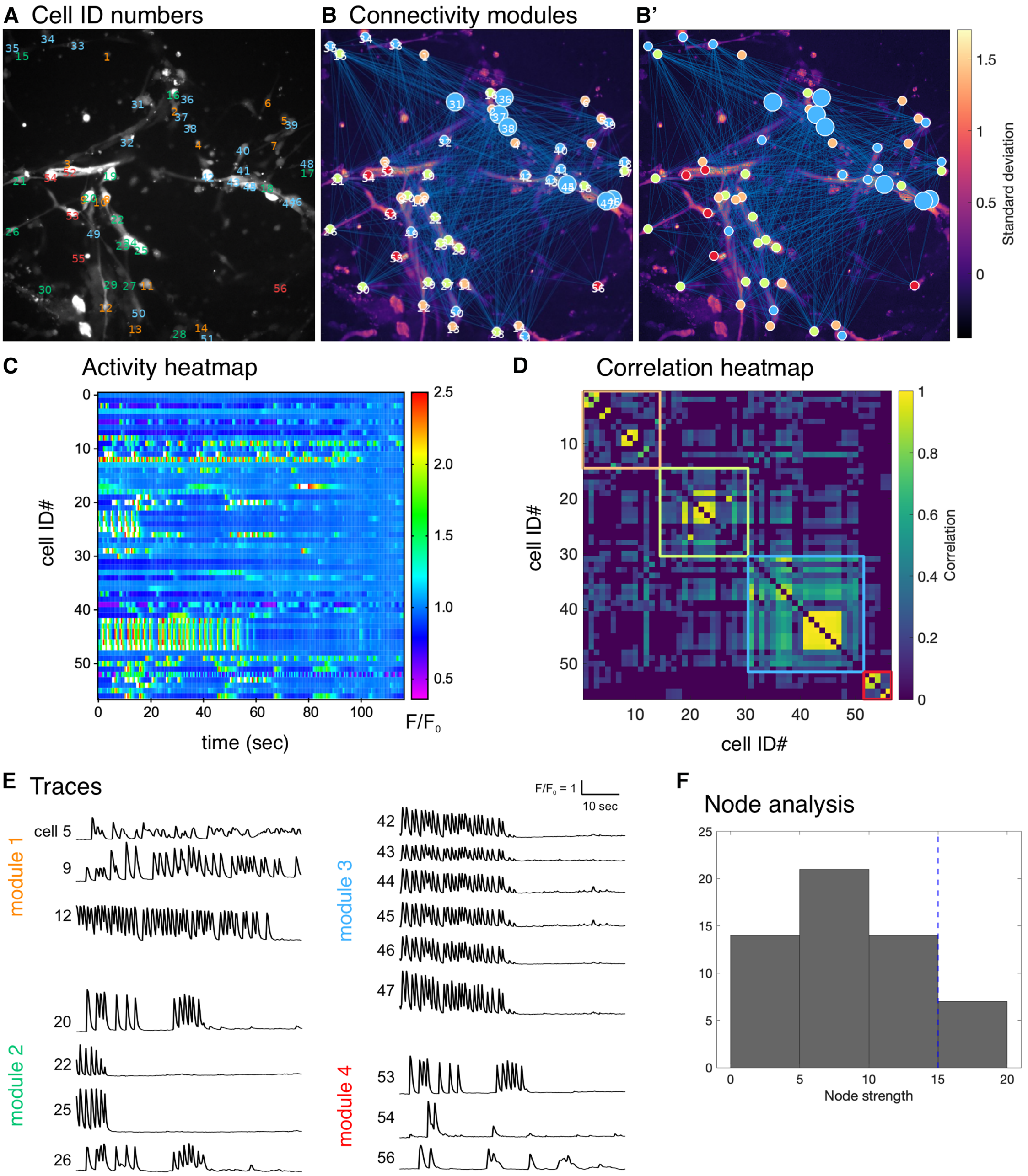

Figure 4. Spontaneous $[Ca^{2+}]_{i,}$ activity at D57 (from WAB-00033)

(A) Fluorescence images of Fluo-4 AM loaded cells at D57 with individual cell ID#s indicated.

(B) Connectivity modules with cell ID# labels added, and excluded (*B′*). Reconstructed neural network based on “graph theory” with blue dots representing nodes and blue lines representing edges, superimposed on a heatmap of the calcium signals (standard deviation across the time series). Nodes are color-coded according to network modules. Larger circles indicate “hub nodes”—neurons with higher connectivity (node strength >40) and a stronger contribution to the overall network connectivity.

(C) Heatmap of $[Ca^{2+}]_{i,}$ activity in individual cells, showing fluorescence intensity after normalization to baseline fluorescence (F/F_0).

(D) Correlation matrix representation of the neural network, where both the *x* and *y* axes correspond to the nodes (cell ID#s), and each element represents the strength of connectivity (correlation value) between pairs of nodes. Matrix elements are arranged according to the modular structure of the network and modules with stronger connectivity are dispersed across the network.

(E) Example traces of $[Ca^{2+}]_{i,}$ activity in individual cells from each module.

(F) Histogram of the degree of each node, emphasizing that 12.5% of neurons play a critical role in network communication. The blue dashed line denotes the threshold applied to define “hub nodes” with high connectivity depicted in (D).

Suárez et al., 2021). We found that modules were not constrained by spatial proximity, but instead were dispersed across distant locations of the network (Figures 4B and S6), indicating long-range crosstalk and functional organization between cells. In addition, 12.5% of neurons were identified as hub nodes, i.e., cells with disproportionally high connectivity to the rest of the network. Network properties could not be evaluated at D22 or D43 due to the sparse activity observed at these stages. At D57, hub nodes were detected in all three recordings where robust spontaneous activity was observed, highlighting the generation of network activity (Figures 4 and S6). Hubs are hypothesized to be central to the functioning of the network, acting as mediators of communication between distant cell groups (Schröter et al., 2017; Sporns et al., 2007) (Figure 4F).

DISCUSSION

Functional activity in enteric neurons is accompanied by changes in intracellular Ca^{2+} dynamics. To investigate the formation of a functional neurocircuitry in iPSC-derived enteric neurons, we investigated gene expression patterns and performed cellular resolution Ca^{2+} imaging for spontaneous and evoked Ca^{2+} transients. Our results show that at all stages of differentiation, a population of cells responds to neurotransmitter agonists and elevated dynamics of K^{+}, indicating the presence of a functional neuronal population. Additionally, cells develop spontaneous neural activity and signatures of neuronal maturation throughout the differentiation program.

In human fetal ENS tissue, evoked Ca^{2+} transients are first observed at embryonic week (EW) 16, despite the presence of dense neuronal networks and expression of excitatory neurotransmitters as early as EW12 (McCann et al., 2019). It is unknown when spontaneous Ca^{2+} activity emerges in the human ENS, although it is not present at EW16. Given that colonization of the human foregut by migratory ENCCs begins at EW4 and is completed by EW7, there is a latent period between the termination of colonization and the emergence of coordinated electrical activity (Fu et al., 2004; Wallace and Burns, 2005). This differs from the mouse, where it has been shown that developing enteric neurons exhibit spontaneous Ca^{2+} transients and respond to exogenous neurotransmitter receptor agonists and electrical field stimulation at embryonic day (E) 11.5 (Hao et al., 2011), the day after they enter the small intestine and two days before they complete colonization (Kapur et al., 1992). In our study, enteric neurons generated from iPSCs exhibited evoked Ca^{2+} activity but little spontaneous Ca^{2+} activity at D22 of differentiation, suggesting that these cells functionally resemble fetal cells aged approximately EW16 (McCann et al., 2019). This accelerated differentiation compared to the *in utero* timeline likely reflects fundamental differences between *in vitro* and *in vivo* maturation contexts, where the application of successive factors *in vitro* is optimized to promote neuronal identity. However, following initial differentiation, a key limitation of iPSC technology is that derivates are typically maintained in an immature state, resembling fetal phenotypes rather than adult cells (Cerneckis et al., 2024). This is in part due to the absence of dynamic and reciprocal biochemical signals, and cell-to-cell communications, that result from the presence of multiple cell types existing in highly complex 3D structures *in vivo*.

The alterations in both amplitude and duration of Ca^{2+} responses observed in our study suggest that iPSC-derived enteric neurons acquire more regulated Ca^{2+} dynamics with prolonged *in vitro* maturation. Our previous studies have shown that the amplitude of evoked $[Ca^{2+}]_i$ transients and action potentials increase during embryonic murine ENS development, peak during early postnatal ages, and then decrease in adults, which is most likely due to further maturation of neurons during postnatal development (Hao et al., 2011; 2012). This pattern of maturation was also observed in human iPSC-derived cells in this study, with increasing evoked $[Ca^{2+}]_i$ transient amplitude from D22 to D43, and then reduction from D43 to D57. One interpretation of the current data could be that cells at D57 are becoming more mature and responses are more refined. The appearance of S100B+ enteric glia at D57 could also support this. Further, the shortening of $[Ca^{2+}]_i$ transient duration observed after D29 is also consistent with previously observed reduction of action potential durations during ENS maturation (Hao et al., 2012). However, how this *in vitro* timeline fits with the acquisition of mature neuronal function in human ENS development is currently unknown. Also, this reduction in $[Ca^{2+}]_i$ amplitude could also arise due to deterioration of neuronal health due to the increased density and detachment of cells in prolonged culture. Interestingly, these Ca^{2+} signatures were only observed following high K^{+} or electrical stimulation and not in response to neurotransmitter receptor agonism. This is in line with studies in mice, where the amplitude of the intracellular Ca^{2+} response following ATP and DMPP stimulus has no obvious correlation with the age of mice (Hao et al., 2011).

Our gene expression and immunohistochemistry data indicate that the number of enteric glia in iPSC-derived enteric cultures increases over time, which is in accordance with previous studies (Barber et al., 2019; Majd et al., 2025). Gene expression signatures of key enteric glial cell markers were synonymous with the *in vivo* development of the mouse enteric nervous system, where enteric glial progenitors first express FABP7 and then sequentially acquire the mature glial markers S100B and

GFAP (Young et al., 2003). For Ca^{2+} imaging experiments, the vast quantity of cells in culture limited the feasibility of identifying individual cells following post hoc immunohistochemistry, meaning that the individual Ca^{2+} response of a cell could not be corroborated with the expression of enteric neuron or glial markers. It is well documented that enteric glia engage in bidirectional communication with enteric neurons to maintain gastrointestinal homeostasis. One of the most characterized forms of enteric neuron to glia communication is mediated by the stimulation of purinergic P2Y1 receptors (P2Y1R) located on the enteric glial cell membrane by ATP or adenosine diphosphate (ADP) (Gomes et al., 2009; Gulbransen and Sharkey, 2009; McClain et al., 2014). Following activation, enteric glia exhibit an increase in intracellular calcium and subsequently release gliotransmitters such as ATP through membrane situated connexin (GJA1) hemichannels. Although we were unable to determine the presence of glial Ca^{2+} transients, gene expression analysis showed expression of *P2Y1R* at all stages of differentiation, indicating that the machinery necessary for enteric glia to respond to purinergic signals is likely present. The emergence of spontaneous calcium activity by D57 may reflect both the progressive maturation of enteric neurons and increasing functional contributions from glial cells. The delayed upregulation of glial markers such as S100B and GFAP suggests that glial maturation occurs later in culture, potentially coinciding with the development of spontaneous network activity. Enteric glia are known to modulate neuronal signaling, maintain extracellular ion balance, and influence synaptic development, all of which could facilitate the emergence of coordinated calcium transients (Gonzales and Gulbransen, 2025). Future experiments employing glial-specific calcium reporters, or selectively manipulating glial populations, will be important for determining the extent to which neuron-glia interactions contribute to the spontaneous activity observed in these cultures.

The success of cell-based therapies for enteric neuropathy will rely heavily on the functionality of the cells being transplanted. It is crucial that transplanted cells are not only alive but also active and capable of proper communication with one another, and the host system. Our study provides insight into the temporal progression of neuronal network formation in iPSC-derived ENS cells. At the 2-month time point, graph theory network analysis provided evidence of neuronal network development, indicating that some level of functional synaptic communication had begun to occur. It showed long-range coordinated activity that appeared to be mediated by hub nodes of neurons, representing just 12.5% of the total responding population. In the brain, these hubs are thought to be central to the functioning of the network, acting as mediators of communication between distant cell groups (Schröter et al., 2017; Sporns et al., 2007). Whether this is also the case in the native ENS is unknown, but our data provide some tantalizing new insights into ENS network dynamics.

In the case of Hirschsprung disease, a condition characterized by the absence of ganglion cells in the colon, the need for a functional transplant that encompasses all required subtypes of enteric neurons and glia is particularly pressing. The challenge of replacing the entire ENS in the colon is considerable due to the complex and diverse roles different neuronal and glial subtypes play in regulating gastrointestinal motility and other functions. Recent studies using Hirschsprung disease mouse models have demonstrated the potential for successful transplantation of hPSC-derived enteric neurons, with survival and integration of transplanted cells, offering promise for therapeutic interventions (Fan et al., 2023; Fattahi et al., 2016). However, further research is needed to understand how networks comprised of diverse neuronal and glial subtypes mature *in vivo*, and whether they exhibit characteristic features of functional ENS activity. The use of graph theory-based network analysis could provide a powerful tool for assessing the functional integration of transplanted neurons, helping to determine whether they establish appropriate synaptic connections and contribute to coordinated activity within host circuits.

Beyond cell transplantation studies, the generation of hPSC-derived enteric neurons and glia offers an opportunity for the development of reliable disease models for gastrointestinal disorders, as well as for the screening of potential therapeutic compounds. While the etiology of Hirschsprung disease is well established, there are a number of debilitating gut disorders, including slow transit constipation, whose origins are poorly understood and may be related to aberrant network connectivity or activity. Better understanding of ENS networks could provide insight into the biological mechanisms underlying a range of gastrointestinal motility disorders. In addition, understanding how different genetic mutations or environmental perturbations affect network maturation will be critical for advancing personalised medicine approaches and guiding regenerative treatments aimed at repairing or replacing damaged ENS cells. This knowledge could inform the identification of novel biomarkers for ENS dysfunction and provide deeper insights into the underlying molecular mechanisms that govern normal and pathological gastrointestinal activity.

RESOURCE AVAILABILITY

Lead contact

Further information and requests for resources and reagents can be directed to and will be fulfilled by the lead contact, Marlene M. Hao (hao.m@unimelb.edu.au).

Materials availability
This study did not generate any new unique reagents.

Data and code availability
This study did not generate new datasets. Raw data and images are available upon request to the corresponding authors.

ACKNOWLEDGMENTS

We thank Professor Pieter Vanden Berghe (KU Leuven, Belgium) for kindly providing the source code for conducting calcium imaging analysis in this work. We also thank members of the Stamp/Hao and Pébay labs at the University of Melbourne for their help with experiments. This study was funded by the NHMRC (2021360) and MRFF (2009049; 2032801). E.S.R. is funded by an Australian government RTP scholarship; A.P. is supported by an NHMRC Senior Research Fellowship (1154389) and a Dame Kate Campbell Fellowship; L.A.S. is funded by NHMRC MRFF grants (2009049; 2032801). M.M.H. was funded by an ARC fellowship (DE190101209) and the NHMRC (2021360).

AUTHOR CONTRIBUTIONS

Conceptualization, M.M.H. and L.A.S.; methodology, E.S.R., F.F., M.D., and A.P.; investigation, E.S.R. and G.S.Y.; analysis, E.S.R., C.S., and M.D.B.; writing – original draft preparation, E.S.R.; writing – review and editing, M.M.H. and L.A.S.; supervision, M.M.H., L.A.S., and M.D.; funding acquisition, M.M.H. and L.A.S. All authors have read and agreed to the published version of the manuscript.

DECLARATION OF INTERESTS

The authors declare no competing interests.

STAR★METHODS

Detailed methods are provided in the online version of this paper and include the following:

- KEY RESOURCES TABLE
- EXPERIMENTAL MODEL AND STUDY PARTICIPANT DETAILS
  - Maintenance of iPSCs
- METHOD DETAILS
  - Differentiation of iPSC into enteric neurons
  - Immunocytochemistry
  - Image acquisition and analysis
  - Gene expression
  - Live-cell calcium imaging
- QUANTIFICATION AND STATISTICAL ANALYSIS
  - Graph theory and network construction
  - Data and statistics

SUPPLEMENTAL INFORMATION

Supplemental information can be found online at https://doi.org/10.1016/j.stemcr.2026.102942.

Received: June 16, 2025
Revised: May 6, 2026
Accepted: May 6, 2026
Published: June 4, 2026

REFERENCES

Barber, K., Studer, L., and Fattahi, F. (2019). Derivation of enteric neuron lineages from human pluripotent stem cells. Nat. Protoc. *14*, 1261–1279. https://doi.org/10.1038/s41596-019-0141-y.

Blondel, V.D., Guillaume, J.-L., Lambiotte, R., and Lefebvre, E. (2008). Fast unfolding of communities in large networks. J. Stat. Mech. *2008*, P10008. https://doi.org/10.1088/1742-5468/2008/10/P10008.

Boesmans, W., Martens, M.A., Weltens, N., Hao, M.M., Tack, J., Cirillo, C., and Vanden Berghe, P. (2013). Imaging neuron-glia interactions in the enteric nervous system. Front. Cell. Neurosci. *7*, 183. https://doi.org/10.3389/fncel.2013.00183.

Bullmore, E., and Sporns, O. (2009). Complex brain networks: graph theoretical analysis of structural and functional systems. Nat. Rev. Neurosci. *10*, 186–198. https://doi.org/10.1038/nrn2575.

Burns, A.J., Goldstein, A.M., Newgreen, D.F., Stamp, L., Schäfer, K.-H., Metzger, M., Hotta, R., Young, H.M., Andrews, P.W., Thapar, N., et al. (2016). White paper on guidelines concerning enteric nervous system stem cell therapy for enteric neuropathies. Dev. Biol. *417*, 229–251. https://doi.org/10.1016/j.ydbio.2016.04.001.

Cerneckis, J., Cai, H., and Shi, Y. (2024). Induced pluripotent stem cells (iPSCs): molecular mechanisms of induction and applications. Signal Transduct. Target. Ther. *9*, 112. https://doi.org/10.1038/s41392-024-01809-0.

Daniszewski, M., Senabouth, A., Liang, H.H., Han, X., Lidgerwood, G.E., Hernández, D., Sivakumaran, P., Clarke, J.E., Lim, S.Y., Lees, J.G., et al. (2022). Retinal ganglion cell-specific genetic regulation in primary open-angle glaucoma. Cell Genom. *2*, 100142. https://doi.org/10.1016/j.xgen.2022.100142.

Fairman, C.L., Clagettdame, M., Lennon, V.A., and Epstein, M.L. (1995). Appearance of neurons in the developing chick gut. Developmental Dynamics *204*, 192–201.

Fan, Y., Hackland, J., Baggiolini, A., Hung, L.Y., Zhao, H., Zumbo, P., Oberst, P., Minotti, A.P., Hergenreder, E., Najjar, S., et al. (2023). hPSC-derived sacral neural crest enables rescue in a severe model of Hirschsprung's disease. Cell Stem Cell *30*, 264–282.e9. https://doi.org/10.1016/j.stem.2023.02.003.

Fattahi, F., Steinbeck, J.A., Kriks, S., Tchieu, J., Zimmer, B., Kishinevsky, S., Zeltner, N., Mica, Y., El-Nachef, W., Zhao, H., et al. (2016). Deriving human ENS lineages for cell therapy and drug discovery in Hirschsprung disease. Nature *531*, 105–109. https://doi.org/10.1038/nature16951.

Foong, J.P.P., Hirst, C.S., Hao, M.M., McKeown, S.J., Boesmans, W., Young, H.M., Bornstein, J.C., and Vanden Berghe, P. (2015). Changes in nicotinic neurotransmission during enteric nervous system development. J. Neurosci. *35*, 7106–7115. https://doi.org/10.1523/jneurosci.4175-14.2015.

Foong, J.P.P., Nguyen, T.V., Furness, J.B., Bornstein, J.C., and Young, H.M. (2012). Myenteric neurons of the mouse small intestine undergo significant electrophysiological and morphological changes during postnatal development. J. Physiol. (Lond.) *590*, 2375–2390. https://doi.org/10.1113/jphysiol.2011.225938.

Frith, T.J.R., Gogolou, A., Hackland, J.O.S., Hewitt, Z.A., Moore, H.D., Barbaric, I., Thapar, N., Burns, A.J., Andrews, P.W., Tsakiridis, A., and McCann, C.J. (2020). Retinoic Acid Accelerates the Specification of Enteric Neural Progenitors from In-Vitro-Derived Neural Crest. Stem Cell Rep. *15*, 557–565. https://doi.org/10.1016/j.stemcr.2020.07.024.

Fu, M., Tam, P.K.H., Sham, M.H., and Lui, V.C.H. (2004). Embryonic development of the ganglion plexuses and the concentric layer structure of human gut: a topographical study. Anat. Embryol. *208*, 33–41. https://doi.org/10.1007/s00429-003-0371-0.

Furness, J.B. (2012). The enteric nervous system and neurogastroenterology. Nat. Rev. Gastroenterol. Hepatol. *9*, 286–294. https://doi.org/10.1038/nrgastro.2012.32.

Gomes, P., Chevalier, J., Boesmans, W., Roosen, L., Van den Abbeel, V., Neunlist, M., Tack, J., and Vanden Berghe, P. (2009). ATP-dependent paracrine communication between enteric neurons and glia in a primary cell culture derived from embryonic mice. Neuro Gastroenterol. Motil. *21*, 870.e62. https://doi.org/10.1111/j.1365-2982.2009.01302.x.

Gonzales, J., and Gulbransen, B.D. (2025). The Physiology of Enteric Glia. Annu. Rev. Physiol. *87*, 353–380. https://doi.org/10.1146/annurev-physiol-022724-105016.

Gulbransen, B.D., and Sharkey, K.A. (2009). Purinergic Neuron-to-Glia Signaling in the Enteric Nervous System. Gastroenterology *136*, 1349–1358. https://doi.org/10.1053/j.gastro.2008.12.058.

Gwynne, R.M., and Bornstein, J.C. (2007). Synaptic transmission at functionally identified synapses in the enteric nervous system: Roles for both ionotropic and metabotropic receptors. Curr. Neuropharmacol. *5*, 1–17. https://doi.org/10.2174/157015907780077141.

Hackland, J.O.S., Frith, T.J.R., Thompson, O., Marin Navarro, A., Garcia-Castro, M.I., Unger, C., and Andrews, P.W. (2017). Top-Down Inhibition of BMP Signaling Enables Robust Induction of hPSCs Into Neural Crest in Fully Defined, Xeno-free Conditions. Stem Cell Rep. *9*, 1043–1052. https://doi.org/10.1016/j.stemcr.2017.08.008.

Hao, M.M., Bergner, A.J., Hirst, C.S., Stamp, L.A., Casagranda, F., Bornstein, J.C., Boesmans, W., Vanden Berghe, P., and Young, H.M. (2017). Spontaneous calcium waves in the developing enteric nervous system. Dev. Biol. *428*, 74–87. https://doi.org/10.1016/j.ydbio.2017.05.018.

Hao, M.M., Boesmans, W., Van den Abbeel, V., Jennings, E.A., Bornstein, J.C., Young, H.M., and Vanden Berghe, P. (2011). Early Emergence of Neural Activity in the Developing Mouse Enteric Nervous System. J. Neurosci. *31*, 15352–15361. https://doi.org/10.1523/jneurosci.3053-11.2011.

Hao, M.M., Foong, J.P.P., Bornstein, J.C., Li, Z.L., Vanden Berghe, P., and Boesmans, W. (2016). Enteric nervous system assembly: Functional integration within the developing gut. Dev. Biol. *417*, 168–181. https://doi.org/10.1016/j.ydbio.2016.05.030.

Hao, M.M., Lomax, A.E., McKeown, S.J., Reid, C.A., Young, H.M., and Bornstein, J.C. (2012). Early Development of Electrical Excitability in the Mouse Enteric Nervous System. J. Neurosci. *32*, 10949–10960. https://doi.org/10.1523/jneurosci.1426-12.2012.

Hendler, B.J., McClain, J.L., Zilli, A., Seguella, L., and Gulbransen, B.D. (2025). Purinergic P2Y(1) and P2Y(12) receptors control enteric nervous system activity through neuro-glia-macrophage crosstalk. Purinergic Signal. *21*, 1033–1048. https://doi.org/10.1007/s11302-024-10060-9.

Heuckeroth, R.O. (2018). Hirschsprung disease — integrating basic science and clinical medicine to improve outcomes. Nat. Rev. Gastroenterol. Hepatol. *15*, 152–167. https://doi.org/10.1038/nrgastro.2017.149.

Kapur, R.P., Yost, C., and Palmiter, R.D. (1992). A transgenic model for studying development of the enteric nervous system in normal and aganglionic mice. Development *116*, 167–175.

Le Douarin, N.M., and Teillet, M.A. (1973). The migration of neural crest cells to the wall of the digestive tract in avian embryo. J. Embryol. Exp. Morphol. *30*, 31–48.

Lee, G., Chambers, S.M., Tomishima, M.J., and Studer, L. (2010). Derivation of neural crest cells from human pluripotent stem cells. Nat. Protoc. *5*, 688–701. https://doi.org/10.1038/nprot.2010.35.

Lee, G., Kim, H., Elkabetz, Y., Al Shamy, G., Panagiotakos, G., Barberi, T., Tabar, V., and Studer, L. (2007). Isolation and directed differentiation of neural crest stem cells derived from human embryonic stem cells. Nat. Biotechnol. *25*, 1468–1475. https://doi.org/10.1038/nbt1365.

Leembruggen, A.J.L., Yildiz, G.S., Hardee, J.P., Stamp, L.A., Bornstein, J.C., and Hao, M.M. (2025). Plasticity of enteric neurotransmission varies during day-night cycles and with feeding state. Am. J. Physiol. Gastrointest. Liver Physiol. *328*, G145–G151. https://doi.org/10.1152/ajpgi.00286.2024.

Majd, H., Samuel, R.M., Cesiulis, A., Ramirez, J.T., Kalantari, A., Barber, K., Farahvashi, S., Ghazizadeh, Z., Majd, A., Chemel, A.K., et al. (2025). Engrafted nitrergic neurons derived from hPSCs improve gut dysmotility in mice. Nature *645*, 158–167. https://doi.org/10.1038/s41586-025-09208-3.

McCann, C.J., Alves, M.M., Brosens, E., Natarajan, D., Perin, S., Chapman, C., Hofstra, R.M., Burns, A.J., and Thapar, N. (2019). Neuronal Development and Onset of Electrical Activity in the Human Enteric Nervous System. Gastroenterology *156*, 1483–1495.e6. https://doi.org/10.1053/j.gastro.2018.12.020.

McClain, J., Grubišić, V., Fried, D., Gomez-Suarez, R.A., Leininger, G.M., Sévigny, J., Parpura, V., and Gulbransen, B.D. (2014). Ca^{2+} responses in enteric glia are mediated by connexin-43 hemichannels and modulate colonic transit in mice. Gastroenterology *146*, 497–507.e1. https://doi.org/10.1053/j.gastro.2013.10.061.

McKeown, S.J., Stamp, L., Hao, M.M., and Young, H.M. (2013). Hirschsprung disease: a developmental disorder of the enteric nervous system. WIREs Dev. Biol. *2*, 113–129. https://doi.org/10.1002/wdev.57.

Montalva, L., Cheng, L.S., Kapur, R., Langer, J.C., Berrebi, D., Kyrklund, K., Pakarinen, M., de Blaauw, I., Bonnard, A., and Gosain, A.

(2023). Hirschsprung disease. Nat. Rev. Dis. Primers *9*, 54. https://doi.org/10.1038/s41572-023-00465-y.

Rubinov, M., and Sporns, O. (2010). Complex network measures of brain connectivity: Uses and interpretations. Neuroimage *52*, 1059–1069. https://doi.org/10.1016/j.neuroimage.2009.10.003.

Schröter, M., Paulsen, O., and Bullmore, E.T. (2017). Micro-connectomics: probing the organization of neuronal networks at the cellular scale. Nat. Rev. Neurosci. *18*, 131–146. https://doi.org/10.1038/nrn.2016.182.

Sporns, O., and Betzel, R.F. (2016). Modular Brain Networks. Annu. Rev. Psychol. *67*, 613–640. https://doi.org/10.1146/annurev-psych-122414-033634.

Sporns, O., Honey, C.J., and Kötter, R. (2007). Identification and Classification of Hubs in Brain Networks. PLoS One *2*, e1049. https://doi.org/10.1371/journal.pone.0001049.

Suárez, L.E., Richards, B.A., Lajoie, G., and Misic, B. (2021). Learning function from structure in neuromorphic networks. Nat. Mach. Intell. *3*, 771–786. https://doi.org/10.1038/s42256-021-00376-1.

Wallace, A.S., and Burns, A.J. (2005). Development of the enteric nervous system, smooth muscle and interstitial cells of Cajal in the human gastrointestinal tract. Cell Tissue Res. *319*, 367–382. https://doi.org/10.1007/s00441-004-1023-2.

Yntema, C.L., and Hammond, W.S. (1954). The origin of intrinsic ganglia of trunk viscera from vagal neural crest in the chick embryo. J. Comp. Neurol. *101*, 515–541.

Young, H.M., Bergner, A.J., and Müller, T. (2003). Acquisition of neuronal and glial markers by neural crest-derived cells in the mouse intestine. J. Comp. Neurol. *456*, 1–11.

STAR★METHODS

KEY RESOURCES TABLE

REAGENT or RESOURCE	SOURCE	IDENTIFIER
**Antibodies**		
Human anti-HuC/D (1:5000)	Gift from Prof. Vanda Lennon, (Fairman et al., 1995)	N/A
Goat anti-hSox10 (1:500)	R&D Systems	Cat# AF2864; RRID: AB_442208
Rabbit anti-S100β (1:1000)	DAKO	Cat# Z0311; RRID: AB_10013383
Mouse anti-neuronal class III beta-Tubulin (Tuj1) (1:2000)	BioLegend	Cat# 801213; RRID:AB_2313773
Goat anti-mouse IgG Alexa Fluor 488 (1:400)	ThermoFisher Scientific	Cat# A11029; RRID: AB_2534088
Donkey anti-sheep Alexa Fluor 594 (1:100)	Invitrogen	Cat# A11016; RRID: AB_10562537
Donkey anti-human Alexa Fluor 647 (1:200)	Jackson	Cat# 709-605-149; RRID: AB_2340578
Donkey anti-rabbit Alexa Fluor 488 (1:800)	Invitrogen	Cat # A-21206; RRID: AB_2535792
**Chemicals, peptides, and recombinant proteins**		
Recombinant human BMP-4 protein	R&D Systems	Cat # 314-BP
SB431542	R&D Systems	Cat # 1614
CHIR99021	Tocris	Cat # 4423
Retinoic Acid	Sigma-Aldrich	Cat # R2625
Human FGF-basic (FGF-2/bFGF) Recombinant Protein	PeproTech	Cat # 100-18B
GDNF	PeproTech	Cat # 78058
Ascorbic Acid	Thermofisher	Cat # A15613.36
Cultrex Mouse Laminin I	R&D Systems	Cat # 3400-010-02
Human fibronectin	Corning	Cat # 354008
**Critical commercial assays**		
SensiFAST cDNA Synthesis Kit	Bioline	Cat # BIO-65053
QIAcuity Probe PCR Kit	Qiagen	Cat # 250103
**Experimental models: Cell lines**		
Human iPSC line: WAB-00033	Prof Alice Pébay, (Daniszewski et al., 2022)	N/A
Human iPSC line: WAB-00263	Prof Alice Pébay, (Daniszewski et al., 2022)	N/A
**Oligonucleotides**		
Primer *ACTB*	Taqman	Hs01060665_g1
Primer *ACTB*	IDTDNA	Hs.PT.39a.22214847

(*Continued on next page*)

Continued

REAGENT or RESOURCE	SOURCE	IDENTIFIER
Primer *EDNRB*	Taqman	Hs00240747_m1
Primer *FABP7*	Taqman	Hs00950761_g1
Primer *GFAP*	Taqman	Hs00909233_m1
Primer *GJA1*	Taqman	Hs00748445_s1
Primer *HOXB5*	IDTDNA	Hs.PT.58.2318474.g
Primer *P2RY1*	Taqman	Hs00704965_s1
Primer *PHOX2B*	Taqman	Hs00243679_m1
Primer *S100B*	Taqman	Hs00902901_m1
Primer *SOX10*	Taqman	Hs00366918_m1
Primer *SOX2*	Taqman	Hs04234836_s1
Primer *SOX8*	Taqman	Hs00232723_m1
Primer *SOX9*	Taqman	Hs00165814_m1
**Software and algorithms**		
Zen Blue 3.2	Zeiss	N/A
ImageJ	NIH	N/A
GraphPad Prism v6	GraphPad	N/A
IGOR Pro	WaveMetrics	

EXPERIMENTAL MODEL AND STUDY PARTICIPANT DETAILS

Generation of the human iPSC lines WAB-00033 (female, 64 years-old at time of biopsy) and WAB-00263 (male, 64 years-old at time of biopsy) used in this study were previously described, including characterisation of pluripotency (Daniszewski et al., 2022). This research falls under Category 1A of the International Society for Stem Cell Research (ISSCR) guidelines, 2025.

Maintenance of iPSCs

The two iPSC lines were maintained as adherent monolayers on non-tissue culture 6-well plates (Corning), coated with vitronectin XF (40 μL/mL; STEMCELL Technologies) diluted in Cell Adhere Dilution Buffer (STEMCELL Technologies). Cells were cultured in supplemented StemFlex medium (ThermoFisher Scientific) containing penicillin-streptomycin (0.1 mg/mL) with media changes every 2–3 days and weekly passaging with the enzyme free reagent, ReLeSR (STEMCELL Technologies) as per the manufacturer's instructions. Both lines were tested for mycoplasma using the MycoAlert mycoplasma detection kit (Lonza) following the manufacturer's instructions. All cultures were maintained in a humidified incubator at 37°C with 5% CO_2.

METHOD DETAILS

Differentiation of iPSC into enteric neurons

Cell lines were differentiated toward an enteric neuron and glial cell identity using a previously published method with minor modifications (Barber et al., 2019). At 80% confluency, iPSCs were dissociated using ReLeSR and plated onto vitronectin XF coated 6-well non-tissue culture plates at a 5:6 split ratio. Cultures were maintained in StemFlex medium for 2 days, until the cultures formed a near confluent monolayer. Cells were then cultured in Essential 6 medium (E6; STEMCELL Technologies) containing bone morphogenic protein-4 (BMP-4; 1 ng/mL; R&D Systems), SB431542 (10 μM; R&D Systems) and CHIR 99021 (600 μM; Tocris) for two days. On day (D) 2 and D4 of the differentiation, media was switched to E6 medium containing SB431542 (10 μM) and CHIR99021 (1.5 μM), with media changes occurring every

2 days. From D6 to D12, media was changed to E6 medium containing SB431542 (10 μM), CHIR99021 (1.5 μM) and Retinoic Acid (1 μM; Sigma Aldrich) and changed every 2 days. On D12, cultures were detached with Accutase (STEMCELL Technologies) for 30 min at 37°C. Each well was individually collected, centrifuged at 290 g for 1 min, and the supernatant aspirated. Cell pellets were resuspended in Neurobasal Medium (NM; Life Technologies) supplemented with N2 supplement (10 μL/mL; Life Technologies), B27 supplement minus Vitamin A (20 μL/mL; Life Technologies), GlutaMax (10 μL/mL; Life Technologies), and MEM Nonessential Amino Acids (MEM NEAA; 10 μL/mL; Corning) and basic fibroblast growth factor 2 (FGF-2; 10 ng/mL; PeproTech) then transferred to 6-well ultra-low attachment plates (Corning). Thereafter, media was changed every 2 days. On D15 of differentiation, cells were dissociated with Accutase and plated on 6-well plastic plates, glass coverslip dishes (Ibidi) or 8-well chamber slides (ThermoFisher) coated with poly-L-ornithine (PO; 15 μg/mL; Sigma Aldrich), laminin (LN; 2 μg/mL; R&D Systems) and fibronectin (FN; 2 μg/mL; Corning) at a density of 100,000 cells per cm^2. Cells were cultured in NM containing N2 supplement (10 μL/mL), B27 supplement minus Vitamin A (20 μL/mL), GlutaMax (10 μL/mL), MEM NEAA (10 μL/mL) and Ascorbic Acid (100 μM; ThermoFisher Scientific) and glial derived neurotrophic factor (GDNF; 10 ng/mL; Peprotech), with media changes every 2 days.

Immunocytochemistry

Cultures grown on 8-well chamber slide glass coverslips were fixed overnight in 10% neutral buffered formalin at 4°C. The following day, cells were washed 3 times with PBS for 10 min at room temperature then permeabilised in a solution of 0.1% Bovine Serum Albumin (BSA) + 0.1% Triton X-100 for 30 min. Cells were washed 3 times in PBS for 10 min then primary antibodies were incubated in PBS overnight at 4°C. Cells were washed 3 times for 10 min, incubated with secondary antibodies for 2 h at room temperature then washed with PBS 3 times for 10 min. Nuclei were counter-stained with DAPI.

Image acquisition and analysis

Fluorescence images were acquired with a Zeiss LSM 900 (Carl Zeiss) confocal microscope using Zen blue 3.2 software (Carl Zeiss). All images were processed and analyzed using ImageJ software (NIH). Confocal images were obtained using a 10x or 20× objective.

Gene expression

RNA was extracted on D0, D21, D28, D35, D42, D49 and D56 of differentiation. Total RNA was extracted by homogenisation with TRIzol Reagent (Invitrogen). Phase separation was performed by addition of chloroform (Sigma) to the sample. RNA was collected from the aqueous phase and then precipitated in an equal volume of isopropanol for 15 min at 4°C, then centrifuged at 12,200 g for 15 min at 4°C. The pellet was then washed with 80% ethanol, centrifuged at 12,200g for 15 min at 4°C and the ethanol discarded. RNA concentration was determined with the NanoDrop One Microvolume UV-Vis Spectrophotometer (ThermoFisher Scientific). Total RNA was purified using the RNeasy Mini Kit (Qiagen) according to the manufacturer's instructions. Synthesis of complementary DNA (cDNA) was performed using the Bioline Sensifast cDNA synthesis kit (Bioline) following the manufacturer's instructions. Individual master mixes were made up using the QIAcuity Probe PCR kit (Qiagen) following the manufacturer's instructions. Digital PCR (dPCR) was performed on a QIAcuity Digital PCR System (Qiagen) in single reactions using a QIAcuity Nanoplate 8.5k 96-well plate (Qiagen) and TaqMan Gene Expression probes. Target transcripts were also analyzed using PrimeTime qPCR Primer assay primers (Intergrated DNA Technologies, Singapore) and the QX200 droplet digital PCR system (Bio-rad Laboratories, NSW, Australia). The number of positive copies per each gene was normalised to the number of positive copies of the *ACTB* housekeeping gene. RNA was stored at −80°C prior to purification and cDNA synthesis. A total of 6 samples were obtained for each time point, from 6 independent experiments (n = 3 for WAB00033 and n = 3 for WAB00263).

Live-cell calcium imaging

Ca^{2+} imaging was performed on D22, D29, D36, D43, D50 and D57 of differentiation. Changes in intracellular Ca^{2+} concentration $[Ca^{2+}]_i$ were assayed by Fluo-4 AM (Invitrogen) fluorescence imaging as described previously (Hao et al., 2011; Leembruggen et al., 2025). Cells were loaded with the calcium indicator dye Fluo-4 AM (5μM) in HEPES solution (in mM; 149 NaCl; 5 KCl; 1 $MgCl_2$; 2 $CaCl_2$; 10 Glucose; 10 HEPES, with pH adjusted to 7.4 using NaOH) for 20 min at RT, then washed for an additional 20 min with fresh HEPES solution. Dishes were then transferred to a recording stage, where cells were constantly perfused with fresh HEPES solution at room temperature using a gravity-fed electronic valve system (ElveFlow). Fields of view were selected where individual cell bodies could be clearly resolved. This often excluded densely populated areas, where segmentation and quantification of calcium signals were not possible. Fluo-4 AM was excited at

470 nm with an LED (Zeiss Colibri) and its fluorescence emission was collected at 525 nm using an inverted microscope (Axiovert 25, Zeiss) and ×20 (NA 0.5) objective lens. Images were obtained at 50 ms exposure time per frame at 2 Hz using an Axiocam 702 mono camera (Zeiss). Recordings were obtained for a total duration of 1–2 min and consisted of 10 s of baseline measurements, followed by local application of solutions or agonists for between 5 and 10 s. Agonists used included high potassium solution (5 s stimulus; in mM: 78 NaCl, 75 KCl, 10 HEPES, 2 $CaCl_2$, 1 $MgCl_2$, and 10 D-glucose, adjusted to pH = 7.4 using NaOH), 1,1-dimethyl-4-phenylpiperazinium (DMPP; 10 s stimulus; 10 μM; Fluka) and adenosine triphosphate (ATP; 10 s stimulus; 10 μM; Sigma). Each recording was followed by a 3 min drug wash-out period, consisting of perfusion with HEPES. All recordings involving solutions or agonists, as well as spontaneous activity recordings, were performed at 37°C in order to mimic physiological conditions. All agonists were diluted in HEPES solution. Images were collected using Zen Blue software (Zeiss). Due to the non-sterile conditions of cell loading and imaging, cells could not be placed back in culture after calcium imaging for sequential analysis of the same cells.

Videos were analyzed in IGOR PRO (Wavemetrics, Lake Oswego, OR, USA) using custom written macros from Prof Pieter Vanden Berghe (KU Leuven, Belgium). Regions of interest (ROIs) were drawn over the cell body of each responding cell. Fluorescence intensity (F) was calculated for each ROI and normalised to its baseline fluorescence value (F_0), which was determined by averaging fluorescence intensity of the first 10 frames. Changes in intracellular Ca^{2+} concentration ($[Ca^{2+}]_i$) were included in analysis only if the signal had a minimum increase of 10 times the intrinsic noise. For amplitude measurements, the maximum increase in $[Ca^{2+}]_i$ above baseline (F/F_0) was determined. For duration measurements, the time taken for the $[Ca^{2+}]_i$ to decay to 50% of its peak value was determined.

QUANTIFICATION AND STATISTICAL ANALYSIS

Graph theory and network construction

To illustrate crosstalk between neurons, a functional network was reconstructed from $[Ca^{2+}]_i$ at D57. Network nodes were defined based on manually delineated coordinates demarcating neurons with significant Ca^{2+} fluctuations, and network edges were computed as the Pearson's correlation in $[Ca^{2+}]_i$ between each cell pair. The network was thresholded to filter out weak correlations by retaining the top-50% strongest edges (based on the absolute value of the Pearson's correlation). To avoid excessive noise in the data, a minimum of 20 active cells was required to conduct this analysis.

Graph theory analyses were performed to characterize patterns of connectivity across the network. First, we followed established procedures based on the Louvain algorithm to partition the network into modules of strongly connected neurons/nodes (Blondel et al., 2008; Rubinov and Sporns, 2010). These modules represent putative functional communities of neurons that exhibit coordinated activity. Second, we computed a node's strength as the sum of its connection weights, i.e., the sum of the absolute Pearson's correlation associated with its edges. Following previous work, we classified the top 15% nodes with highest strength as hubs (Sporns et al., 2007). Hub nodes exhibit disproportionate connectivity to other network elements and are thought play a central role in integrating information across the network.

Data and statistics

Data are presented as mean ± the standard error of the mean (SEM). For qPCR experiments, cells were pooled from each well for RNA collection, therefore each "*N*" represents a biological replicate. For calcium imaging, each "*n*" represents an individual cell, with data collected from a minimum of 3 biological differentiation replicates for each iPSC line. All statistical analyses and graphical data were generated using Graphpad Prism software (v6, www.graphpad.com). Statistical significance was $p < 0.05$.

Stem Cell Reports, Volume *21*

Supplemental Information

Functional maturation of enteric neurons derived from human induced pluripotent stem cells

Eve S. Rowland, Maciej Daniszewski, Caio Seguin, Maria A. Di Biase, Gunes S. Yildiz, Atefeh Namipashaki, Alice Pébay, Faranak Fattahi, Lincon A. Stamp, and Marlene M. Hao

SUPPLEMENTARY INFORMATION

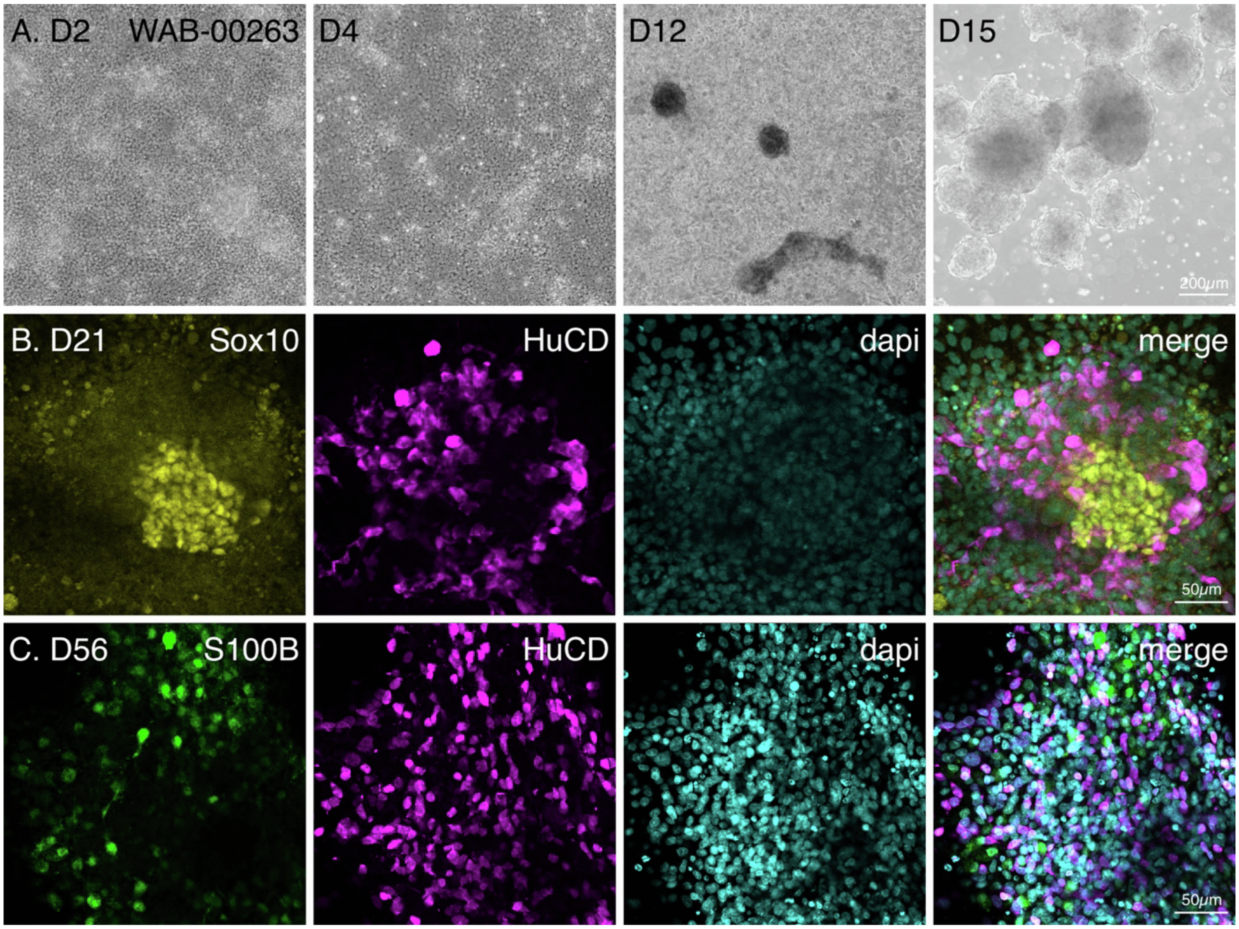

Figure S1: Representative images of differentiation of iPSC-derived ENS for WAB-00263. ***A:*** Representative brightfield images iPSCs at different days of differentiation. ***B:*** iPSCs at D21 of differentiation with immunohistochemistry performed against SOX10 and HuCD. ***C:*** D56 cells with immunohistochemistry against S100B and HuCD. Scale bar = 50 μm.

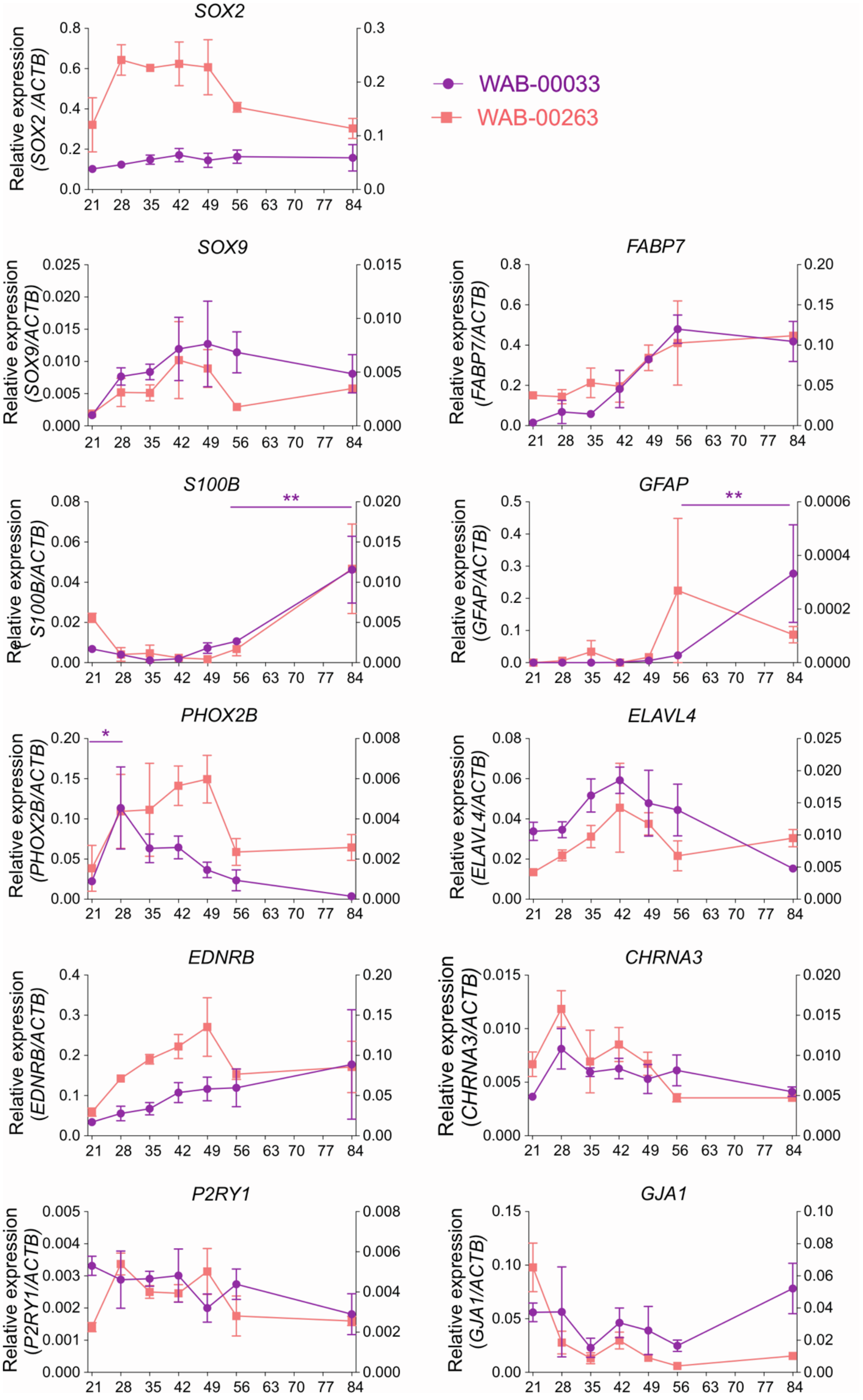

WAB-00033
WAB-00263
SOX2
SOX9
FABP7
S100B
GFAP
PHOX2B
ELAVL4
EDNRB
CHRNA3
P2RY1
GJA1
Relative expression (SOX2 /ACTB)
Relative expression (SOX9/ACTB)
Relative expression (FABP7/ACTB)
Relative expression (S100B/ACTB)
Relative expression (GFAP/ACTB)
Relative expression (PHOX2B/ACTB)
Relative expression (ELAVL4/ACTB)
Relative expression (EDNRB/ACTB)
Relative expression (CHRNA3/ACTB)
Relative expression (P2RY1/ACTB)
Relative expression (GJA1/ACTB)
21 28 35 42 49 56 63 70 77 84
**
*

Figure S2: Quantitative analysis of expression levels of genes typically expressed during ENS development. Including key neural crest genes (*SOX2*, *SOX9*, *EDNRB*, *PHOX2B*); markers of glial differentiation (*FABP7*, *S100B*, *GFAP*), enteric neurons (*ELAV4*), and genes important in ENS communication (*CHRNA3*, *P2RY1*, *GJA1*), in both WAB-00033 *(purple)* and WAB-00263 *(orange)* cell lines over the time-course of differentiation. Statistically significant changes were observed for glial markers *S100B* and *GFAP* between D56 and D84, and also *PHOX2B* between D21 and D28. Two-way ANOVA followed by Tukey's post hoc test, ** $p < 0.01$, $N = 3$ for each cell line.

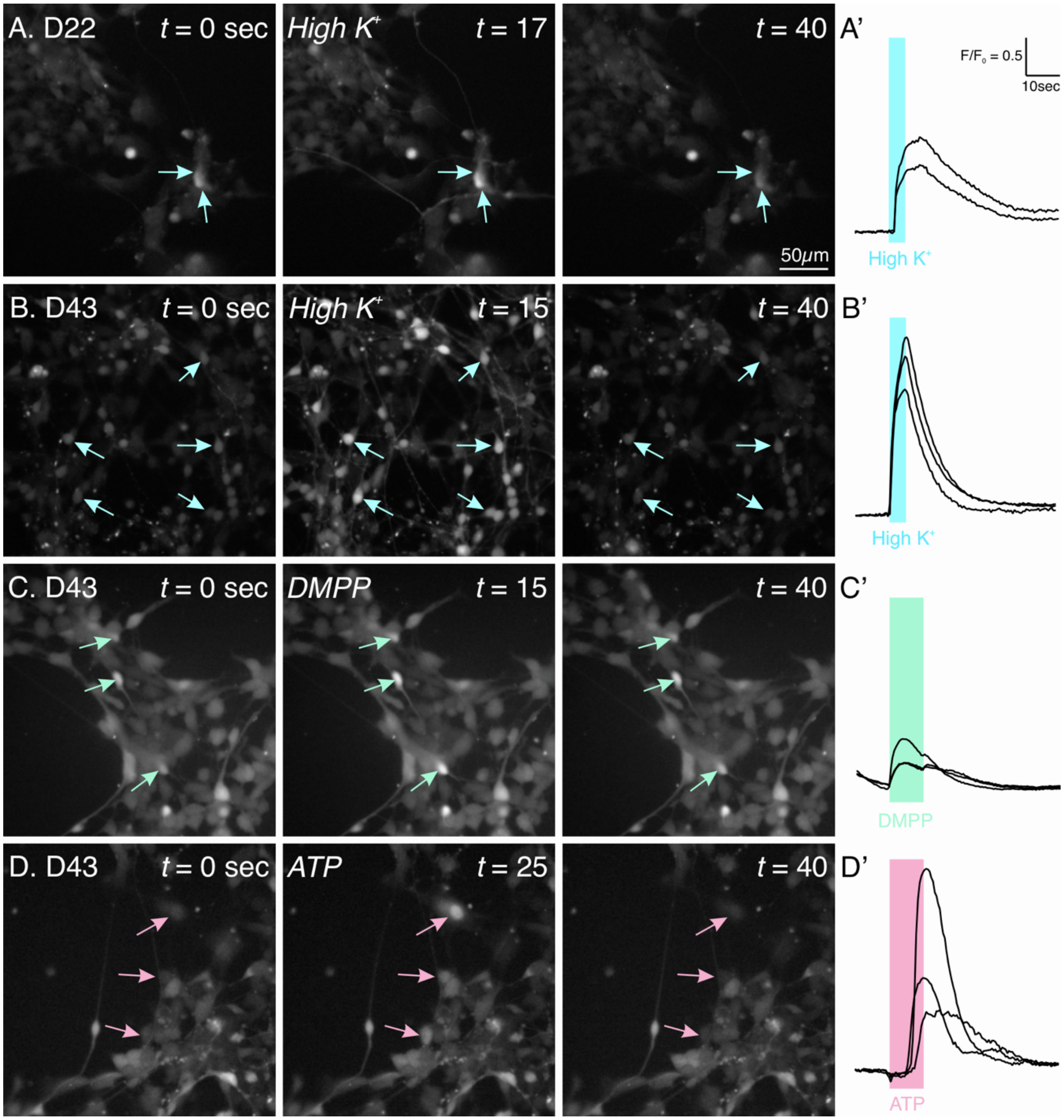

Figure S3: Live Ca^{2+} imaging of iPSC-derived ENS from WAB-00263 in response to depolarisation and neurotransmitter agonists. ***A-B:*** Fluorescence images of Fluo-4 AM loaded cells at D22 ***(A)*** and D43 ***(B)*** at baseline (T = 0 sec), then following High K^+ stimulation (T = 15-17) and (T = 40), with active cells highlighted by arrows. Scale bar: 50 µm. Representative traces of Ca^{2+} response to High K^+ stimulus shown in *A'* and *B'*. Changes in $[Ca^{2+}]_i$ are shown by fluorescence intensity normalised to the baseline fluorescence (F/F_0). Coloured boxes show the duration of High K^+ stimulation from T = 10 – 15 seconds. Vertical scale bar: F/F_0 = 0.5; horizontal scale bar: T = 10 seconds. ***C-D:*** Fluorescence images of Fluo-4 AM loaded cells at D43 at baseline (T = 0 sec), following neurotransmitter agonist stimulation (T = 20) and (T = 40). Scale bar: 50 µm. Representative traces of Ca^{2+} response to DMPP stimulus ***(C,C')*** and ATP stimulus ***(D,D')***.

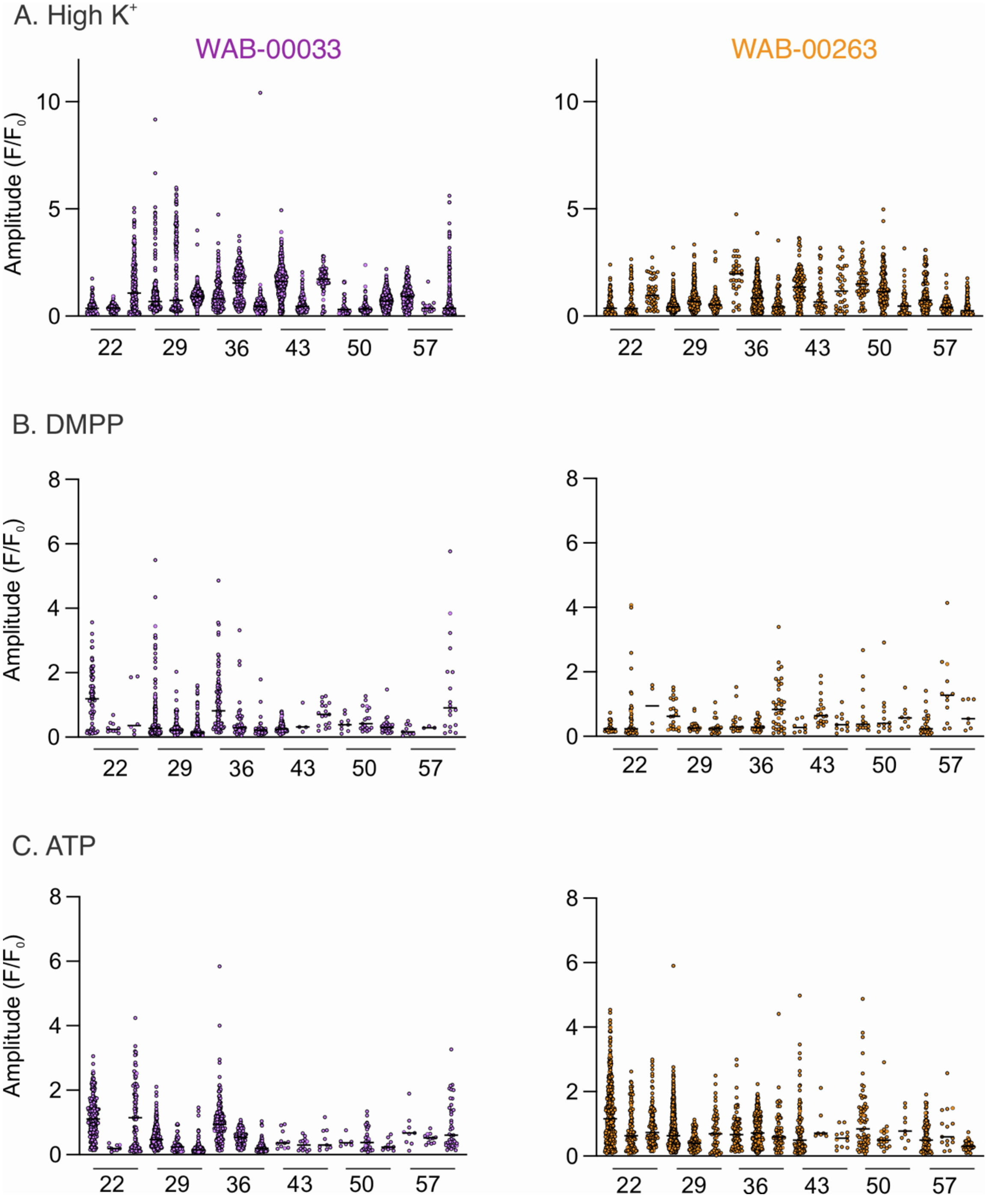

Figure S4: Quantification of evoked $[Ca^{2+}]_i$ transients (F/F_0) in response to High K^+ ***(A)***, DMPP ***(B)*** and ATP ***(C)***, showing individual biological replicates for each cell line: WAB-00033 and WAB-00263. Each data point represents an individual cell, with population means also indicated.

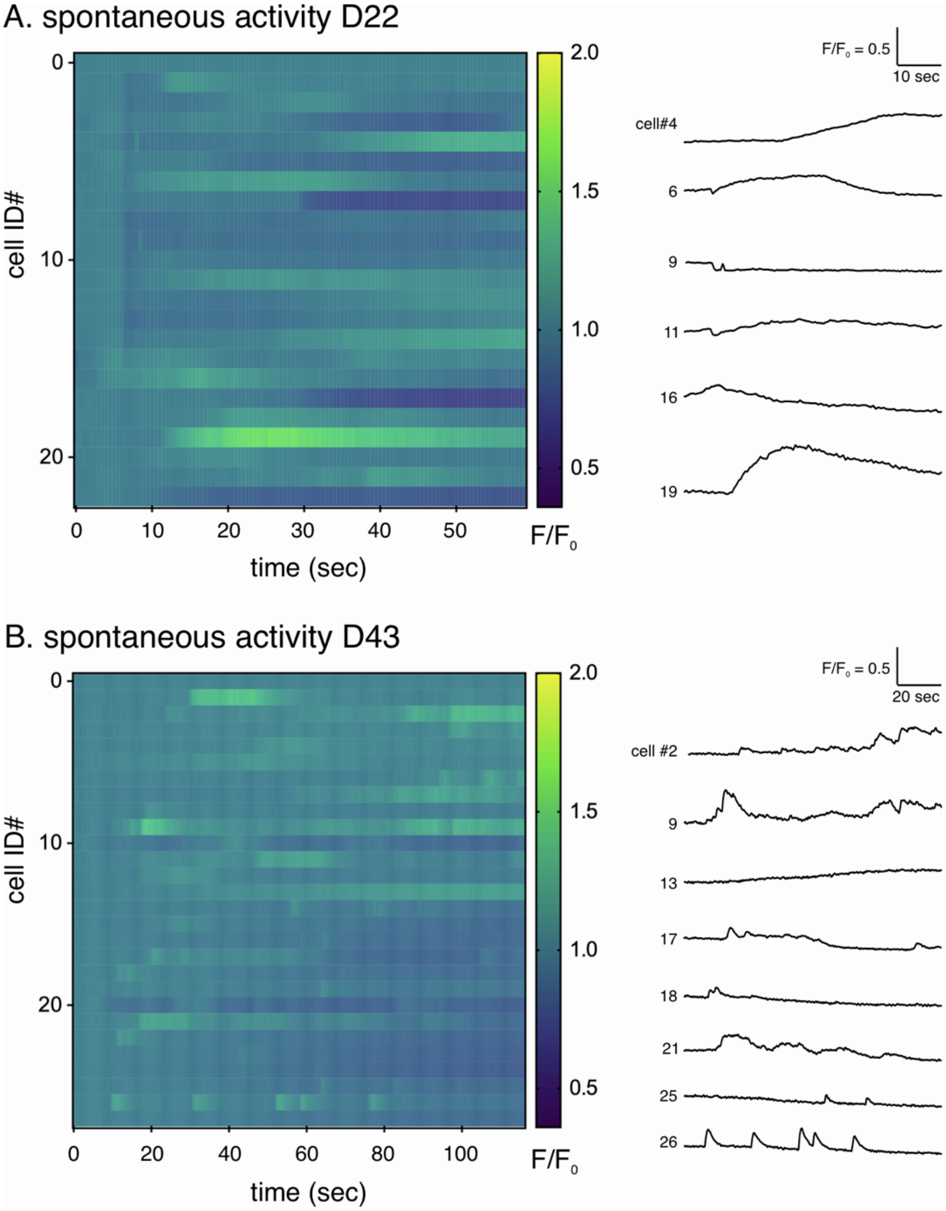

Figure S5: Spontaneous $[Ca^{2+}]_{i,}$ analysis at D22 ***(A)*** and D43 ***(B)***. Heatmap of changes in $[Ca^{2+}]_{i,}$ with fluorescence intensity normalised to the baseline fluorescence F/F_0. Representative traces of spontaneous Ca^{2+} transients from individual cells shown on right. Some spontaneous neuronal $[Ca^{2+}]_{i,}$ transients were present at both D22 and D43, however, activity in individual cells was generally infrequent.

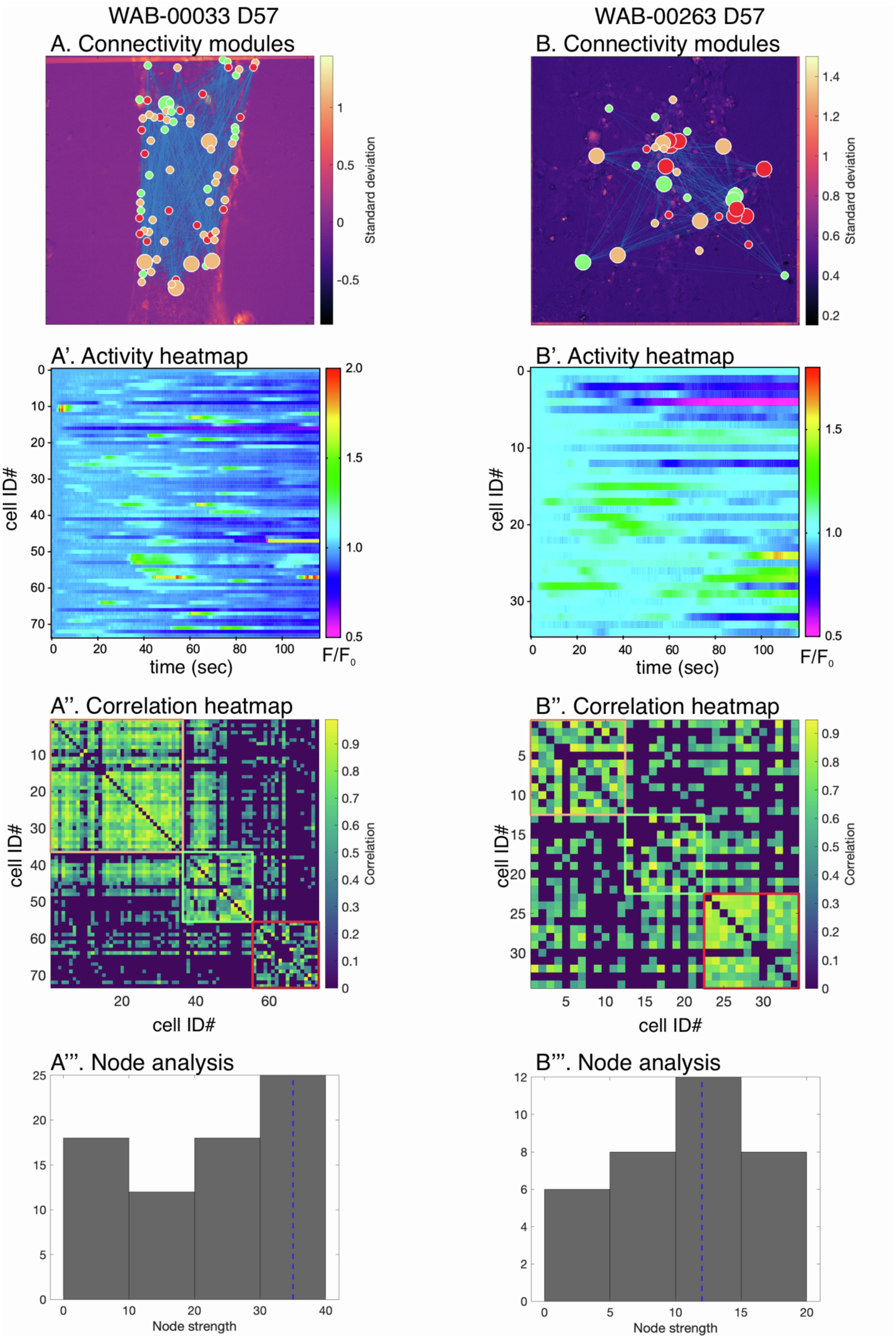

WAB-00033 D57
A. Connectivity modules
Standard deviation
A'. Activity heatmap
cell ID#
time (sec)
F/F0
A''. Correlation heatmap
Correlation
cell ID#
A'''. Node analysis
Node strength
WAB-00263 D57
B. Connectivity modules
Standard deviation
B'. Activity heatmap
cell ID#
time (sec)
F/F0
B''. Correlation heatmap
Correlation
cell ID#
B'''. Node analysis
Node strength

Figure S6: Graph theory analysis of $[Ca^{2+}]_i$ network activity at D57 in two separate recordings, one from WAB-00033 (*left, A-A'''*) and one from WAB-00263 (*right, B-B'''*). ***A,B:*** Connectivity modules for each recording were calculated, reconstructed neural network based on 'graph theory' with blue dots representing nodes and blue lines representing edges superimposed on a heatmap of the calcium signals (standard deviation across the time series). Nodes are colour-coded according to network modules. ***A',B':*** Heatmap of $[Ca^{2+}]_{i,}$ activity in individual cells, showing fluorescence intensity after normalisation to baseline fluorescence (F/F_0). ***A'',B'':*** Correlation matrix representation of the neural network, where both the x and y axes correspond to the nodes (cell ID#s), and each element represents the strength of connectivity (correlation value) between pairs of nodes. ***A''',B''':*** Histogram of the degree of each node. The blue dashed line denotes the threshold applied to define 'hub nodes' with high connectivity depicted in *(A,B)*.
